# Phloroglucinol Treatment Induces Transgenerational Epigenetic Inherited Resistance Against *Vibrio* Infections and Thermal Stress in a Brine Shrimp (*Artemia franciscana*) Model

**DOI:** 10.3389/fimmu.2019.02745

**Published:** 2019-11-27

**Authors:** Suvra Roy, Vikash Kumar, Peter Bossier, Parisa Norouzitallab, Daisy Vanrompay

**Affiliations:** ^1^Laboratory of Immunology and Animal Biotechnology, Department of Animal Sciences and Aquatic Ecology, Faculty of Bioscience Engineering, Ghent University, Ghent, Belgium; ^2^Laboratory of Aquaculture & Artemia Reference Center, Department of Animal Sciences and Aquatic Ecology, Faculty of Bioscience Engineering, Ghent University, Ghent, Belgium; ^3^ICAR-Central Inland Fisheries Research Institute, Barrackpore, India

**Keywords:** brine shrimp, vibrio, transgenerational, epigenetics, innate immunity

## Abstract

Emerging, infectious diseases in shrimp like acute hepatopancreatic necrosis disease (AHPND) caused by *Vibrio parahaemolyticus* and mortality caused by other *Vibrio* species such as *Vibrio harveyi* are worldwide related to huge economic losses in industrial shrimp production. As a strategy to prevent disease outbreaks, a plant-based phenolic compound could be used as a biocontrol agent. Here, using the brine shrimp (*Artemia franciscana*) as a model system, we showed that phloroglucinol treatment of the parental animals at early life stages resulted in transgenerational inherited increased resistance in their progeny against biotic stress, i.e., bacteria (*V. parahaemolyticus* AHPND strain and *V. harveyi*) and abiotic stress, i.e., lethal heat shock. Increased resistance was recorded in three subsequent generations. Innate immune-related gene expression profiles and potential epigenetic mechanisms were studied to discover the underlying protective mechanisms. Our results showed that phloroglucinol treatment of the brine shrimp parents significantly (*P* < 0.05) enhanced the expression of a core set of innate immune genes (*DSCAM, proPO, PXN, HSP90, HSP70*, and *LGBP*) in subsequent generations. We also demonstrated that epigenetic mechanisms such as DNA methylation, m6A RNA methylation, and histone acetylation and methylation (active chromatin marker i.e., H3K4Me3, H3K4me1, H3K27me1, H3 hyperacetylation, H3K14ac and repression marker, i.e., H3K27me3, H4 hypoacetylation) might play a role in regulation of gene expression leading toward the observed transgenerational inheritance of the resistant brine shrimp progenies. To our knowledge, this is the first report on transgenerational inheritance of a compound-induced robust protected phenotype in brine shrimp, particularly protected against AHPND caused by *V. parahaemolyticus* and vibriosis caused by *V. harveyi*. Results showed that epigenetic reprogramming is likely to play a role in the underlying mechanism.

## Introduction

Vibriosis caused by *Vibrio parahaemolyticus* infects various shrimp species and causes acute hepatopancreatic necrosis disease (AHPND), initially named early mortality syndrome (EMS). *V. harveyi* is another pathogen from the Vibrionaceae family that causes substantial mortality by vibriosis and economic loss in shrimp aquaculture (1). Thus, both infections affect animal welfare and are economically devastating to the shrimp industry (2). Shrimp production in affected regions has dropped to ~60%, with up to 100% mortality and led to tremendous global losses estimated at more than $1 billion per year (3, 4). To prevent vibriosis, in particular AHPND/EMS, many conventional approaches such as the use of antibiotics and disinfectants have been applied but had very little success. Antibiotics can no longer be used as feed additives for prevention of infectious diseases (3, 5). Therefore, there is an urgent need to develop innovative disease preventive methods that also support sustainable shrimp aquaculture.

The epigenetic processes that involve transgenerational transfer of phenotypic traits without modifying the gene sequence information have drawn attention of evolutionary biologists and health scientists and could provide important tools, complementary to, e.g., selection for increased disease resistance (6). The term “epigenetics” literally means “above” or “on top of” genetics, and this can be defined as “the study of changes in gene expression/function that are mitotically and/or meiotically heritable and that do not entail a change in DNA sequence” (7, 8). Epigenetic changes involve chromatin remodeling, e.g., DNA methylation, histone modifications, and RNA-based epigenetic regulatory control, i.e., non-coding RNAs (ncRNA) such as microRNAs, small RNAs, and long RNAs (lncRNAs) and RNA methylation (9, 10). Currently, it is well-known that epigenetic programming during the early life stage not only can affect the organism directly in subsequent life stages but also can transmit traits via the germline to subsequent generations in a non-mendelian fashion (11, 12). Additionally, epigenetics reprogramming can be used to train the immune system by pre-exposing them to various stimuli, and it could serve as a promising approach ensuring enhanced immune response and disease resistance in cultured animals. The approach mainly focuses on treating the parental generation with biotic or abiotic environmental stressors resulting in the production of larvae with elevated environmental fitness and disease resistance phenotypes. Therefore, epigenetic programming might be an innovative broodstock management method, complementary to selective breeding (6, 13). For true transgenerational inheritance, the changes in phenotype or memory must pass on at least beyond the F2 generation; hence, F3 will be the first true generation that did not get exposed to the factor/insult itself (14). Brine shrimp (*Artemia franciscana*) can be maintained under axenic conditions, which allows the control of host-associated microbial communities. Well-established vibrio infection models as well as abiotic stress models are available (15, 16). In addition, the *Artemia* genome sequence shares high homology with the genomes of other crustaceans (17). Thus, there is a reasonable chance that results on administration of immunostimulants in axenic brine shrimp can be extrapolated to other crustaceans.

The brine shrimp is also a particularly appropriate model organism to study transgenerational epigenetic inheritance (18–20). Apart from being an established axenic host–pathogen model, brine shrimp are relatively small, they have a very short generation cycle, and hence are easy to handle in animal and laboratory facilities (18). In addition, depending on the environmental conditions (favorable or unfavorable), adults can use two independent reproduction pathways, which allows the production of either encysted gastrula-stage embryos, called “cysts” (dormant eggs) by oviparous reproduction or swimming larvae, called “nauplii” by ovoviviparous reproduction (19) (the life cycle of brine shrimp is explained in Figure S1). Cysts can be stored in the fridge for a couple of years, and after terminating the diapause, cysts of different generations can be hatched simultaneously, permitting to perform a common garden experiment, avoiding or minimizing environmental influences.

Exposure to non-lethal heat shock (NLHS) of shrimp *Penaeus vannamei* (21), green mussel *Perna viridis* (22), and brine shrimp *A. franciscana* (23) induced the expression of heat shock proteins (Hsp70 and Hsp90) and subsequently activated the innate immune system (e.g., proPO system in *Penaeus vannamei*), resulting in enhanced disease resistance against *Vibrio* infections (*V. parahaemolyticus, V. alginolyticus*, and *V. campbellii*). NLHS has been successfully used in parthenogenetic brine shrimp for inducing transgenerational inherited resistance against vibriosis (17). Thus, exposure to NLHS might be a promising strategy for protecting animals and their progenies against vibriosis and possibly other infections. However, its practical application in industrial shrimp farming is cumbersome. In addition, temperature shifts are often detrimental and can negatively affect physiological and immunological balance to the cultured animals (24). Therefore, the administration of heat shock protein (HSP)-inducing compounds as an alternative to NLHS exposure has been proposed (16, 25, 26). Plant-based phenolic compounds, known for their antioxidant/pro-oxidant activities, might be used for this purpose as they can positively affect the immune response and survival of the host (24, 27). Kumar et al. (16) showed that the plant derived polyphenolic compound phloroglucinol might be used as a potential biocontrol agent as it increased the resistance against *V. parahaemolyticus* AHPND infection in gnotobiotic brine shrimp larvae as well as in freshwater shrimp (*Macrobrachium rosenbergii*) larvae by inducing endogenous heat shock protein 70 (Hsp70).

In this study, using brine shrimp as an animal model, we investigated whether phloroglucinol exposure of the parental generation at early life stages could elicit a disease-resistant phenotype in the animals as well as transgenerational inheritance of the acquired disease-resistant phenotype. Hereto, the brine shrimp parental population (TF0) was exposed to phloroglucinol (2 μM) until day after hatching (DAH) 16. Phloroglucinol exposure of the parental generation could induce and transmit a disease-resistant phenotype in three subsequent unexposed generations. Subsequent generations were more resistant against biotic (bacterial challenge) and abiotic (lethal heat shock) stressors. Underlying molecular and epigenetic mechanisms of the observed transgenerational inheritance were examined by studying the expression of innate immune-related genes, measuring global DNA (5-mC) methylation, RNA (m6A) methylation, and histone modifications. To the best of our knowledge, this is the first description of transgenerational inheritance of compound-induced robustness, resulting in protection against both biotic (AHPND strain *V. parahaemolyticus* and *V. harveyi*) and abiotic stressors (thermal stress).

## Materials and Methods

### Culture of Experimental Animals

Brine shrimp (*A. franciscana*) cysts (dormant embryos) originating from the Great Salt Lake in Utah (USA) (EG® type, batch 21452, INVE Aquaculture, Dendermonde, Belgium) were used for production of the parental generation (F0). *Artemia* cysts were hatched in axenic conditions. Briefly, 2 g of *A*. *franciscana* cysts were hydrated in 89 ml of distilled water for 1 h. Sterile larvae were obtained via decapsulation using 3.3 ml of NaOH (32%) and 50 ml of NaOCl (50%), providing 0.2-μm filtered aeration. Decapsulation was stopped after 2 min by adding 50 ml of Na_2_S_2_O_3_ at 10 g/L followed by washing the cysts with filtered autoclaved seawater (FASW) containing 35 g/L of instant ocean synthetic sea salt (Aquarium Systems, Sarrebourg, France). The decapsulated cysts, suspended in 1-L glass bottles containing FASW, were incubated for hatching at 28°C with constant illumination of approximately 27 μE/m^2^ s. After 28 h of incubation, hatched larvae at developmental stage instar II (mouth was opened to ingest food) were collected and used for the experiments.

### Phloroglucinol Treatment of the Parental (F0) Generation

Phloroglucinol (Sigma-Aldrich, Belgium), a polyphenol derivative organic plant-derived compound, was dissolved in sterile distilled water at 0.4 g/L (3.17 mM). Before starting the current experiments, we had tested the protective effect of phloroglucinol in brine shrimp larvae who were continuously exposed to different doses (0.25 to 10 μM) of phloroglucinol and subsequently infected with *V. parahaemolyticus* strain M0904. A dose of 2 μM resulted in a significant protection against mortality as compared to untreated controls.

**Figure 1:**
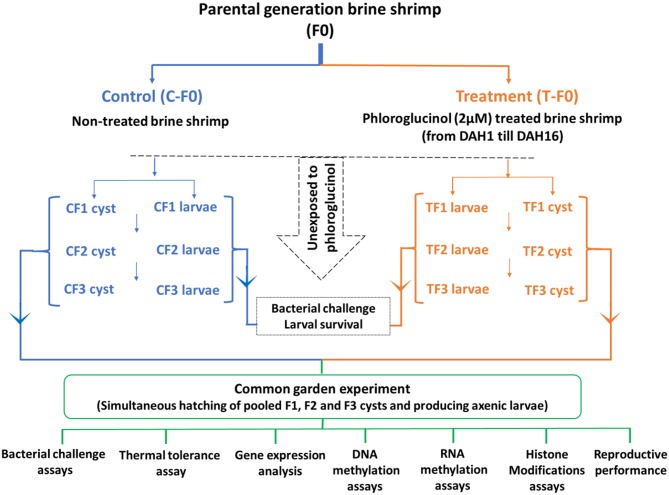
Schematic representation of the experimental setup. For the transgenerational experiment, a total of 2,500 brine shrimp animals in the control and treated population were used. In the treated population, the parental brine shrimp (TF0) were treated with phloroglucinol (2 μM), starting from DAH1 after hatching until DAH16. In the control population, parental shrimp (CF0) were maintained under the same culture and water exchange conditions, but in the absence of phloroglucinol. Adult parental females (CF0 and TF0) produced next-generation CF1 and TF1 larvae and, under suboptimal conditions, CF1 and TF1 cysts. Adult females CF1 and TF1 produced CF2 and TF2 larvae and, under suboptimal conditions, CF2 and TF2 cysts. Subsequently, adult females CF2 and TF2 produced CF3 and TF3 larvae and, under suboptimal conditions, CF3 and TF3 cysts. The F1, F2, and F3 larvae and cysts were never exposed to phloroglucinol. F1, F2, and F3 larvae from treated and untreated parents were subsequently used for *V. parahaemolyticus* and *V. harveyi* challenge assays monitoring survival from 12 h until 60 h post infection. For the common garden experiment, all cysts were hatched simultaneously. Then, age- and size-synchronized axenic 1-day old brine shrimp larvae were used for: (i) *V. parahaemolyticus* and *V. harveyi* challenge assays (7 replicates at the challenge level with 10 larvae/group) and (ii) a thermal tolerance assay (5 replicates at the challenge level with 20 larvae/group). In addition, for each group, 3 replicates at the challenge level with 100 one-day old larvae before and after challenge (6 and 12 h post infection) and 3 replicates of 5 juveniles (16 days old) were sampled for (iii) gene expression analysis, (iv) DNA methylation assays, (v) RNA methylation assays, and (vi) histone modification assays. Finally, FI, F2, and F3 brine shrimp adults (7 pairs) were examined for reproductive performance, namely, the production of cysts and nauplii.

The transgenerational experiment (Figure 1) was performed as previously described (18–20). To collect enough larvae and cyst samples for the whole transgenerational experiment and to be able to perform all the subsequent analysis the parental control population and phloroglucinol-treated population (a total of 2,500 animals/population; animals were distributed in four 2-L glass bottles/population for practical reasons, e.g., easy handling, related to culturing the animals). Animals were cultured in 35 g/L sterilized artificial seawater (28°C, continuous aeration and constant illumination with a light intensity of 27 μmol/m^2^/s). During the entire culture period, all animals were daily *ad libitum* fed with live green algae (*Tetraselmis suecica*). In the treated population, the parental brine shrimp (TF0) were treated with phloroglucinol (2 μM), starting from DAH1 after hatching until DAH16. For this purpose, every 3rd day, water was exchanged, adding fresh phloroglucinol (2 μM). Treatment was stopped at DAH16, to ensure that the uterus, which normally develops by 3 weeks and carries the cysts/embryos, was not directly exposed to the compound. In the control population, parental shrimp (CF0) were cultured in the absence of phloroglucinol and maintained under the same culture and water exchange conditions.

### Production and Collection of Progeny

Adult females of the F0 generation (after 30 days post-hatching) from both control and treatment groups produced larvae (F1 generation). All F1 larvae were further cultured to maturity under controlled environmental conditions as previously explained. F2 larvae were collected from adult F1 brine shrimp. The experiment continued till the F3 generation (Figure 1). F1, F2, and F3 larvae were never exposed to phloroglucinol and later used for a bacterial challenge assay.

Under non-optimal environmental conditions (such as high salinity or low oxygen), brine shrimp (*Artemia*) switches from an ovoviviparous to an oviparous mode of reproduction. Hence, to induce the production of F1, F2, and F3 cysts, the adult F0, F1, and F2 animals in both the control and treatment group were cultured by adding autoclaved artificial seawater with gradually increasing the salinity from 35 to 80 g/L over a 2-week period (Figure 1). The generated F1, F2, and F3 cysts were remained in diapause and were not hatched at that point in time. To terminate the diapause, cysts were conditioned by incubating them in the −20°C freezer for 1 month followed by incubation at 4°C for another 2 months (28). One day after hatching, larvae were used in the common garden experiment.

### Bacterial Strains and Growth Conditions

Two pathogenic strains from the *Vibrio* clade were used: (i) *V. parahaemolyticus* AHPND strain M0904 (29) and (ii) the *Vibrio harveyi* strain BB120 (ATCC BAA-1116) (30). The M0904 strain originated from the Collection of Aquatic Important Microorganism (CAIM) at the A.C. Mazatlàn unit of Aquaculture (Mazatlàn, Sinaloa, Mexico) and was identified as an AHPND strain using an AP3 primer-based PCR (Figure S5). Bacteria were cultured on Luria Bertani agar plates as previously described (30) and 40% glycerol stocks were stored at −80°C until used. For the challenge assays, bacterial strains were cultured overnight in Marine Broth 2216 (Difco Laboratories, Detroit, MI. USA) at 28°C under constant agitation (100 min^−1^). Bacterial cell density was measured spectrophotometrically at 550 nm, taking the McFarland standard into account, assuming that an optical density of 1.0 corresponds to 1.2 × 10^9^ cells/ml. Sterile LVS3 (*Aeromonas hydrophila*) were used as brine shrimp feed during the bacterial challenge assay. An LVS3 stock culture was prepared as previously described (30), and the 24-h-old LVS3 culture was autoclaved (120°C for 20 min), washed in autoclaved artificial seawater, and used at approximately 10^7^ cells/ml.

### Bacterial Challenge Assay

One-day-old brine shrimp larvae from F1 to F3, at each generation from both phloroglucinol treated and non-treated parents, were experimentally infected (immersion, 10^7^ CFU/ml) with *V. parahaemolyticus* (M0904) or *V. harveyi* (BB120) as previously described (16, 24). For each experimental infection at F1 to F3, seven replicates (at the challenge level) of 20 larvae from non-treated parents and 7 replicates (at the challenge level) of 20 larvae from phloroglucinol-treated parents were maintained in glass tubes containing 10 ml of 35 g/L sterile artificial seawater and fed with autoclaved LVS3 (*A. hydrophila*). During the survival assay, the glass tubes with experimentally infected brine shrimp larvae from both the control and treatment group were placed randomly in a rotor and maintained at 6 rotations per minute (rpm). The rotor was placed in the climatized challenge room where the temperature was constantly maintained at 28°C and 2000 lux permanent illumination was present. The survival of the brine shrimp larvae was scored from 12 h post infection onwards till 60 h post infection.

### Common Garden Experiment

CF1 to CF3 cysts and TF1 to TF3 cysts originating from non-treated and treated parents, respectively, were used for the common garden experiment (18, 31). All cysts were hatched simultaneously in axenic conditions for producing age- and size-synchronized larvae as previously described (32). For each group, 7 replicates (at the challenge level) of 10 one-day-old larvae were experimentally infected (10^7^ cells/ml) with *V. parahaemolyticus* (M0904*)* or *V. harveyi* (BB120) and monitored for survival from 12 to 48 h post infection. Also, for each group, 5 replicates (at the challenge level) of 20 one-day-old larvae were exposed to abiotic stress, namely, lethal heat shock (15 min at 42.5°C) and monitored for survival every 3 h post exposure from 0 to 12 h post exposure. During the survival assay as previously described, the glass tubes were placed randomly in a rotor (6 rpm) and placed in the climatized challenge room (temperature 28°C and illumination 2000 lux). In addition, from each group, three replicates (at the challenge level) of 100 one-day-old larvae were sampled for (i) gene expression analyses, (ii) examination of DNA methylation, (iii) examination of RNA methylation, and (iv) studying histone modifications. The later analyses were always performed on larvae sampled before and after challenge (6 and 12 h post infection). Also, 100 remaining 1-day-old larvae from each group were allowed to grow out to juveniles. At the age of 16 days, for each group, 3 replicates of 5 juveniles were sampled for (i) gene expression analyses, (ii) examination of DNA methylation, (iii) examination of RNA methylation, and (iv) studying histone modifications. Sampled larvae and juveniles were frozen in liquid nitrogen and stored at −80°C until analyses. The remaining 40 juveniles per group (F1–F3) were grown to adults and 7 adult pairs were examined for reproduction performance (production of cysts and nauplii).

### RNA Extraction and cDNA Synthesis

Total RNA was extracted from brine shrimp samples (larvae and juveniles) in triplicate (three biological replicates at the challenge level) with Qiagen RNeasy Plus Mini Kit (Cat No. 74136). In each replicate, 100 larvae and 5 juveniles were used for RNA extraction. The quality and quantity of RNA isolates were checked on NanoDrop spectrophotometer (ThermoFisher Scientific, Belgium) and RNA samples with *A*_260_/*A*_280_ ratios >2.0 and *A*_260_/*A*_230_ ratios >1.5 used for the analysis. Reverse transcription was done from 1 μg of total RNA samples with the RevertAid H Minus First Strand cDNA Synthesis Kit (Thermo Fisher Scientific, Belgium) according to the manufacturer's guidelines.

### Standard Curve Preparation for Primer Specificity and Amplification Efficiency

For each set of gene specific primers, the efficiency and specificity were verified via melting curve analysis and standard curves was prepared. Briefly, after PCR amplification, the amplified PCR product was cloned into the plasmid pGEMT vector and then sequenced to confirm the qPCR primers of all genes. Dilution series (1:10) of all the cloned plasmid were prepared, and a standard curve was drawn by plotting the Ct (threshold cycle) values against the logarithm of the dilution factors. The slope of the regression line is related to the amplification efficiency (“*E*”) was calculated for each gene by *E* = *b*^(−1/m)^, where “*b*” is the base of the logarithm.

### Quantitative Real-Time PCR (RT-qPCR) Analysis

Expressions of 10 target immune-related genes, heat shock protein 70 (hsp70), heat shock protein 90 (hsp90), down syndrome cell adhesion molecule (DSCAM), lipopolysaccharide and β-1,3-glucan-binding protein (Lgbp), prophenoloxidase (proPO), high mobility group box 1 (HMGB1), peroxinectin (PXN), superoxide dismutase (SOD), transglutaminase 1 (TGase1), and transglutaminase 2 (TGase2), were measured by RT-qPCR with pair of specific primers (Table S1) using StepOnePlus Real-time PCR systems (Applied Biosystems). EF-1a and GAPDH, which were previously identified as most stably expressed reference genes in brine shrimp were used for qPCR normalization (33). The Ct values from the two reference genes, elongation factor-1alpha (EF-1a) and glyceraldehyde-3-phosphate dehydrogenase (GAPDH), were subjected to geomean and used as the internal control. The amplification was performed in a total volume of 20 μl, containing 10 μl of 2X Maxima SYBR Green/ROX qPCR Master Mix (Thermo Fisher Scientific), 1 μl of cDNA (50 ng), 7 μl of nuclease free water, and 1 μl of each specific primer. Master mix was prepared for three biological replicates and two technical replicates for each sample. RT-qPCR for target and reference genes was performed with a four-step amplification protocol: initial denaturation (10 min at 95°C), 40 cycles of amplification and quantification (15 s at 95°C, 30 s at 60°C, and 30 s at 72°C), melting curve (55–95°C with a heating rate of 0.10°C/s and a continuous fluorescence measurement), and cooling (4°C). Negative control reaction was included for each primer set by omitting template cDNA. The comparative CT method (2^−ΔΔCt^ method) following Livak and Schmittgen (34) was used to analyze the expression level of the target genes and verified by Pfaffl relative standard curve method (35). The log-transformed 2^ΔΔCT^ values were subjected to analysis of *t*-test, and the *p*-values smaller than 0.05 were considered statistically significant. For the interpretation of results, the relative immune gene expression with at least >1.5 at 1% level of significance and >2.0 at 5% level of significance was considered. The heatmap was generated using heatmapper online tool http://heatmapper.ca/.

### DNA Extraction

Genomic DNA was extracted from cysts (100 cysts/replicate), larvae (100 larvae/replicate), and juveniles (5 juvenile/replicate) of F0, F1, F2, and F3 generations of brine shrimp in triplicates using the Qiagen DNeasy Blood & Tissue Kit (Cat No. 69504) according to the manufacturer guidelines. The quality and quantity of purified DNA were checked using the NanoDrop spectrophotometer (ThermoFisher Scientific, Belgium). DNA samples with *A*_260_/*A*_280_ ratios between 1.8 and 2.0 and *A*_260_/*A*_230_ ratios >2.0 were used for further analysis.

### Global DNA and RNA Methylation

Global DNA methylation was determined by using the MethylFlash Global DNA Methylation (5-mC) ELISA Easy Kit (Colorimetric) (P-1030, Epigentek, USA). The methylated fraction of DNA was quantified using 5-methylcytosine (5-mC)-specific antibodies and the amount of methylated DNA is proportional to the optical density (OD) measured at an absorbance of 450 nm in an ELISA microplate spectrophotometer (Tecan Infinite M200 Microplate Reader). In brief, a standard curve was generated by plotting the OD values of the positive control (PC) at each percentage point in defined dilutions, and slope of the standard curve was calculated. From all samples, i.e., brine shrimp cysts, larvae and juveniles of F0, F1, F2, and F3 generations, 100 ng DNA was used for analysis in triplicates.

According to the manufacturer's guidelines, the following formula was used to calculate the percentage of methylated DNA (5-mC) in the total DNA:

5-mC% = (Sample OD – NC OD)/(Slope × *S*) × 100%, where *S* is the amount of input sample DNA (100 ng), OD is the optical density at 450 nm, and NC stands for the negative control. Genomic DNA methylation levels were expressed as 5-mC%.

N6-methyladenosine (m6A) in RNA was measured using the EpiQuick m6A RNA Methylation Quantification Kit (colorimetric) (P-9005, Epigentek, USA) following the manufacturer's instructions and also previous studies (36–39). In brief, a standard curve was prepared by the PC (provided by the kit) at six different concentrations from 0.01 to 0.5 ng/μl. Total RNA was extracted (using Qiagen RNeasy Plus Mini Kit as above) from all the brine shrimp control and treatment sample cysts (100 cysts/replicate), larvae (100 larvae/replicate), and juveniles (5 juveniles/replicate) and 3 biological replicates used for analysis. Total RNA (200 ng) from brine shrimp samples (treatment and control) and negative control (provided by kit) were added to the strip wells. m6A was measured using capture and detection antibodies following the manufacturer's instructions. The detected signal was enhanced and m6A content was quantified colorimetrically by reading the OD value in ELISA microplate spectrophotometer at a wavelength of 450 nm (Tecan Infinite M200 Microplate Reader). The percentage of m6A on total RNA was calculated using the following formula provided by the manufacturer:

m6A (ng) = (Sample OD – NC OD)/Slope and m6A % = m6A Amount (ng)/*S* × 100%. In this equation, NC stands for negative control, PC stands for positive control, and *S* represents the amount of input RNA: 200 ng.

### Histone Extraction

Total histone was extracted from brine shrimp controls and treated cysts (3 replicates with 100 cysts/replicate), larvae (3 replicates with 100 larvae/replicate), and juveniles (3 replicates with 5 juveniles/replicate). Briefly, the brine shrimp samples, i.e., cysts (after decapsulation), larvae, and juveniles were washed twice with PBS in 1.5 ml of protein LoBind Eppendorf tube at 1,000 × *g* for 5 min. Next, the samples were homogenized (until no clumps left in solution) and incubated overnight in 0.4 N HCl on a rotor at 4°C to promote the lysis of nuclei and solubilization of histones. The extract was centrifuged at 16,000 × *g* for 10 min, and then the supernatant was transferred to a new protein LoBind Eppendorf tube. Next, trichloroacetic acid (TCA) (Sigma-Aldrich) was added (drop by drop) to the supernatant, and the tubes were mixed for several times until it turns to a milky solution and afterwards incubated on ice for 2 h to promote the precipitation of histones. The precipitated histones were centrifuged at 16,000 × *g* for 10 min and pelleted histone was washed twice with ice-cold acetone to remove the TCA. Finally, histone pellet was air dried and resuspended in nuclease-free water. Histone concentration was determined following the Bradford method using bovine serum albumin as standard (40) and subsequently the histone quality was checked in 4–20% SDS-PAGE gel using calf thymus histone as control (Figure S7).

### Histone H3 and H4 Total Acetylation and Multiple Modifications by ELISA

Histone H3 and H4 total acetylation were analyzed from 7- to 16-day brine shrimp parental generation and three subsequent F1, F2, F3 generations (cysts and juveniles). Briefly, 1 μg of histone samples both from control and treatment brine shrimp was used to examine the H3 and H4 total acetylation in an ELISA-based format using Histone H3 Total Acetylation Detection Fast Kit (ab131561, abcam, UK) and Histone H4 Total Acetylation Detection Fast Kit (ab131562, abcam, UK), respectively, in triplicate following the manufacturer's instructions.

Next, the histone H3 and H4 multiple modifications in brine shrimp cysts of F1, F2, and F3 generation from both treatment and control were screened using EpiQuik™ Histone H3 Modification Multiplex Assay Kit (P-3100, Epigentek, USA) and EpiQuik™ Histone H4 Modification Multiplex Assay Kit (P-3102, Epigentek, USA) according to the manufacturer's instructions. In brief, 200 ng of histone extracts (two biological replicates) was used to determine the histone H3 and H4 modification at specific sites by a capture antibody that was coated in strip wells that targets the appropriate histone modification pattern and detected with a detection antibody. Colorimetric data were obtained from Tecan Infinite M200 Microplate Reader and OD values were measured at 450 nm wavelength and 655 nm as reference wavelength for background subtraction. H3 and H4 modifications were calculated following the manufacturer's instructions, which also accounts for total histone protein amounts, and the final values for each modification were presented as percentage over control.

H3 or H4 Modification or total H3 or H4 (ng/μg protein) = (sample OD – blank OD) ÷ *S* × 1000/(Assay Control OD – Blank OD) ÷ *P*, where *S* is the amount of input sample protein in ng and *P* is the amount of input assay control in ng.

H3 or H4 Modification % = Amount of H3 or H4 modification (ng/μg protein)/Amount of total H3 or H4 (ng/μg protein) × 100%.

Relative Change % = H3 or H4 modification % in treated sample/H3 or H4 modification % in control sample × 100%.

### Protein Extraction

Stored (−80°C) parenteral brine shrimp (7 and 16 days old, 10 individuals) were homogenized in cold buffer K (150 mM sorbitol, 70 mM potassium gluconate, 5 mM MgCl_2_, 5 mM NaH_2_PO_4_, and 40 mM HEPES, pH 7.4) supplemented with a protease inhibitor cocktail (Sigma-Aldrich, USA). Afterwards, samples were centrifuged (2,200 × *g* for 1 min at 4°C) and the protein concentrations in the supernatants were measured by the Bradford method (40) using a bovine serum albumin (Carl Roth, Germany) as a standard curve.

### Western Blotting

For Hsp70 analysis, protein extracts from the parents (7 and 16 days old) were combined with loading buffer, vortexed, heated for 5 min at 95°C, and then electrophoresed in 10% SDS-PAGE gel (BioRad, Belgium) with each lane receiving equivalent amounts of protein (5 μg). Protein extracted from a suspension of heat-shocked HeLa cells (Enzo Life Sciences, USA) (6 μg) served as positive control. Separated proteins were transferred to a polyvinylidene fluoride membrane (BioRad Immuno-BlotTM PVDF) for antibody probing. Membranes were incubated with blocking buffer [50 ml of 1 × phosphate-buffered saline containing 0.2% (v/v) Tween 20 and 5% (w/v) bovine serum albumin] at room temperature for 60 min. All antibody dilutions were made in Tris-saline and all washing steps were performed using Tris–saline buffer. For Hsp70 analysis, membranes were incubated with a mouse monoclonal anti-Hsp70 antibody (3A3) (1/5000, overnight; Affinity BioReagents Inc., Golden, CO) and horseradish peroxidase-conjugated donkey anti-mouse IgG (1/2500, 2 h; Affinity BioReagents Inc., Golden, CO).

For histone analysis, 5 μg of total histone extracts from F1, F2, and F3 cysts were electrophoresed on a 4–20% SDS-PAGE gel (BioRad, Belgium); histone calf thymus (Roche, 10223565001) (2 μg) served as a positive control. Separated proteins were transferred to a polyvinylidene fluoride membrane (BioRad Immuno-BlotTM PVDF) for antibody probing using (i) rabbit anti-histone H3 trimethyl K4 immunoglobulin (1/1000, overnight; ab8580) in combination with goat anti-rabbit IgG (H + L) (1/1500, 2 h; ab205718), (ii) rabbit anti-histone H3 trimethyl K9 immunoglobulin (1/1000, overnight; ab8898) in combination with goat anti-rabbit IgG (H + L) (1/1500, 2 h; ab205718), and (iii) a mouse monoclonal antibody against histone H3 trimethyl K27 (1/1000, overnight; ab6002) in combination with goat anti-mouse IgG (H + L) (1/1500, 2 h; ab205719). The membranes were then incubated with clarity Western ECL substrate (chemiluminescence reagent) (BioRad Laboratories) for 5 min and the signals were detected by a ChemiDoc MP imaging system (BioRad, Belgium). The relative signal intensity was quantified using Bio-Rad Image lab 4.1 software.

### Statistical Analysis

Survival data were analyzed by logistic regression analysis using GenStat 16 (VSN International, Hemel Hempstead, UK) software to determine significant differences between the control and treatment group. For each time point, least significant difference (LSD) values were generated at different *p* levels. Survival differences between the control and treatment at each time point were tested for the significance based on LSD at different *p* levels. Results for the gene expressions were represented as fold-changes relative to the geometrical mean of two internal control genes (EF-1a and GAPDH). The expression level in the control was regarded as 1.0 and thereby the expression ratio of the treatments was expressed in relation to the control. Analysis for significant differences in expression levels between the control and treatment groups was performed with the single-tailed Student's *t*-tests using log-transformed data, and a two-way analysis of variance (ANOVA) was also performed for the interaction analysis using the Statistical Package for the Social Sciences (SPSS) 19.0 (IBM, Armonk, NY, USA). Significant differences between the control and the treatment group for DNA and m6A RNA methylation, total histone H3 and H4 acetylation, and histone multiplex modifications at each generation were determined by the Student's *t*-test using SPSS version 20.0 (IBM, Armonk, NY, USA). The significance level was set at *P* < 0.05.

## Results

### Phloroglucinol Treatment of Parental Brine Shrimp Increased the Resistance of Their Progeny for Three Subsequent Unexposed Generations

#### Against Bacterial Infection With *V. parahaemolyticus* or *V. harveyi*

**Figure 2:**
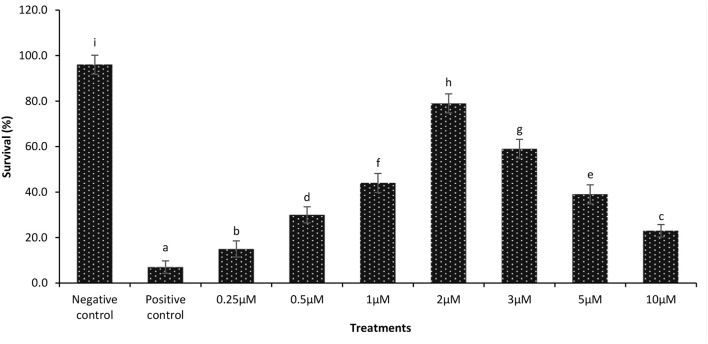
Dose response data on survival of 1-day-old axenic brine shrimp larvae after continuous exposure to different concentrations of phloroglucinol. Phloroglucinol treatment of axenic brine shrimp larvae protected them significantly against a subsequent *V. parahaemolyticus* M0904 challenge (10^7^ cells/ml). Survival percentages of phloroglucinol-treated brine shrimp larvae 48 h post infection are presented. Phloroglucinol untreated larvae that were either challenged with *V. parahaemolyticus* (positive control) or unchallenged (negative control) served as controls. Error bars represent the standard deviation (*n* = 5). Different letters indicate significant differences (*P* < .001).

During a previously performed dose response experiment, it was verified that a continuous exposure of gnotobiotic brine shrimp to 2 μM phloroglucinol could confer protection against *V. parahaemolyticus-*induced mortality (79% increase in survival as compared to untreated shrimp) (Figure 2). Next, we examined if the protective effect induced by phloroglucinol could be passed on to the next brine shrimp generations. For this purpose, an experiment was conducted in which only the parental generation of brine shrimp (F0) was treated (TF0) with phloroglucinol (2 μM), and then, unexposed brine shrimp larvae of the subsequent generations (F1, F2, and F3, produced ovoviviparously), were experimentally infected with *V. parahaemolyticus* M0904 or *V. harveyi* BB120.

**Figure 3:**
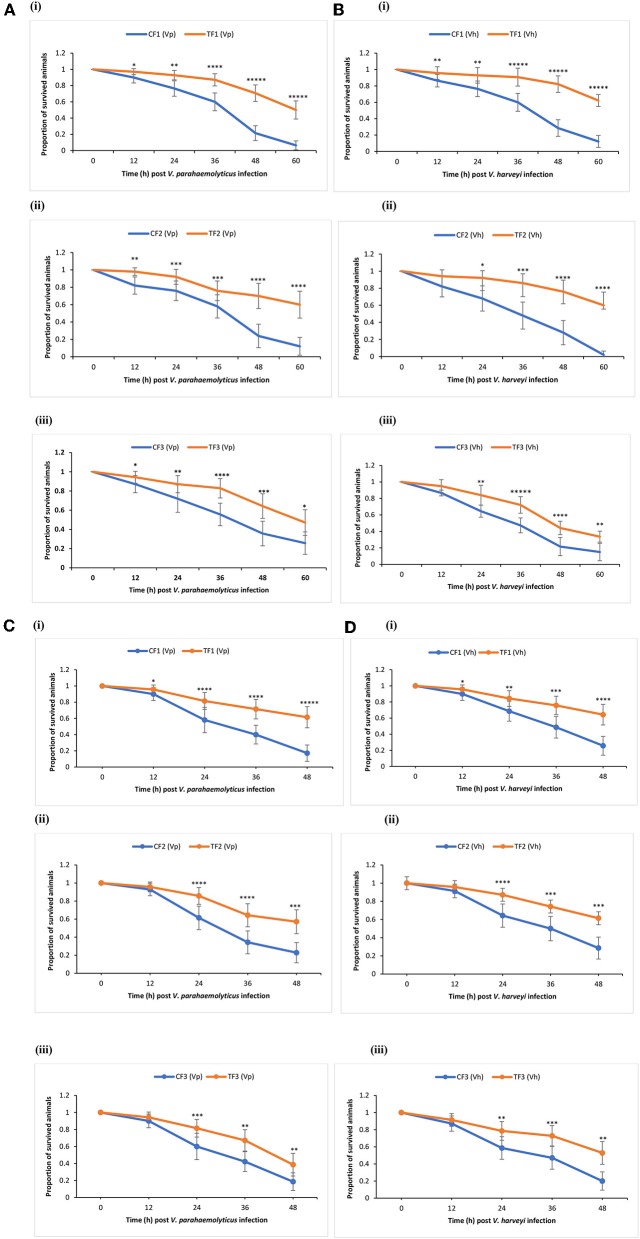
Phloroglucinol treatment of parental brine shrimp increased the resistance of their progeny for three subsequent generations against a bacterial infection with *Vibrio parahaemolyticus* or *Vibrio harveyi*. The parental generation treatment population (TF0) was treated with phloroglucinol (2 μM) until DAH16 and then three subsequent generations TF1, TF2, and TF3 were kept unexposed to phloroglucinol. The parental generation control population (CF0) as well as three subsequent generations CF1, CF2, and CF3 were not treated with phloroglucinol (see Figure 1 for detailed explanations). F1, F2, and F3 larvae from treated and untreated parents were subsequently used for vibrio (10^7^ cells/ml) challenge trials scoring the survival from 12 until 60 h post challenge with *Vibrio parahaemolyticus* (AHPND strain M0904) **(A)** or *Vibrio harveyi* (strain BB120) **(B)**. During the subsequent common garden experiment, age- and size-synchronized 1-day-old larvae were used for vibrio (10^7^ cells/ml) challenge trials scoring the survival from 12 until 48 h post challenge with *Vibrio parahaemolyticus* (AHPND strain M0904) **(C)** or *Vibrio harveyi* (strain BB120) **(D)**. Error bars represent the standard deviation (*n* = 7) and stars represent the significant difference over time at each time point (*P < 0.05, **P < 0.01, ***P < 0.001, ****P < 0.0001, *****P < 0.00001) between the treatment groups and the control groups.

TF1 brine shrimp whose parents (TF0) were exposed to phloroglucinol showed significant higher survival rates following challenge test with *V. parahaemolyticus* or *V. harveyi* as compared to their respective (CF1) controls (Figures 3Ai,Bi). Interestingly, increased resistance toward *V. parahaemolyticus*- or *V. harveyi*-induced mortality was also transmitted to TF2 and TF3 brine shrimp (Figures 3Aii,iii,B ii,iii). These findings were confirmed and validated during a common garden experiment under gnotobiotic conditions to minimize environmental influence (Figures 3Ci–iii,Di–iii). Therefore, age- and size-synchronized larvae were used, which were obtained by hatching the oviparous produced brine shrimp cysts (which were collected from the F0–F2 animals from both control and treatment groups) under axenic conditions, allowing to challenge gnotobiotic F1–F3 larvae with bacteria being *V. parahaemolyticus* or *V. harveyi*. Together, our results showed that phloroglucinol treatment of parental brine shrimp (F0) at an early life stage increased the resistance of their progeny for three subsequent unexposed generations (F1, F2, and F3) against a bacterial infection with *V. parahaemolyticus* or *Vibrio harveyi* at the end of the monitoring period.

#### Against Lethal Heat Shock

Western blotting was used to measure Hsp70 protein production in brine shrimp from parents (TF0; on the 7th and 16th DAH), which were treated by continuous exposure to phloroglucinol (2 μM). Exposure to phloroglucinol significantly increased Hsp70 production (~2-fold) on DAH16 as compared to the control (CF0) (Figure S2). Next, resistance against lethal heat shock (42.5°C for 15 min) was examined for brine shrimp animals during a common garden experiment by simultaneously hatching the cysts of F1, F2, and F3. TF1 to TF3 animals all displayed a significantly higher thermotolerance as compared to CF1 to CF3 controls (Figures S3a–c). Our findings indicate that phloroglucinol treatment of parental brine shrimp at early life stages induced transgenerational inherited thermotolerance at the end of the monitoring period.

Animals originating from phloroglucinol-treated parents (TF0) suffered no reproduction cost or changes in reproductive behavior. There were no significant differences (*P* > 0.05) in total offspring production (cysts and nauplii) by TF1 to TF3 generations in comparison to total offspring production by their respective controls (CF1 to CF3) (Figures S4A–C). Thus, phloroglucinol treatment of the parental generation had no influence on reproduction during three subsequent generations.

### Transcription of Innate Immune-Related Genes in F1–F3 Brine Shrimp

**Figure 4:**
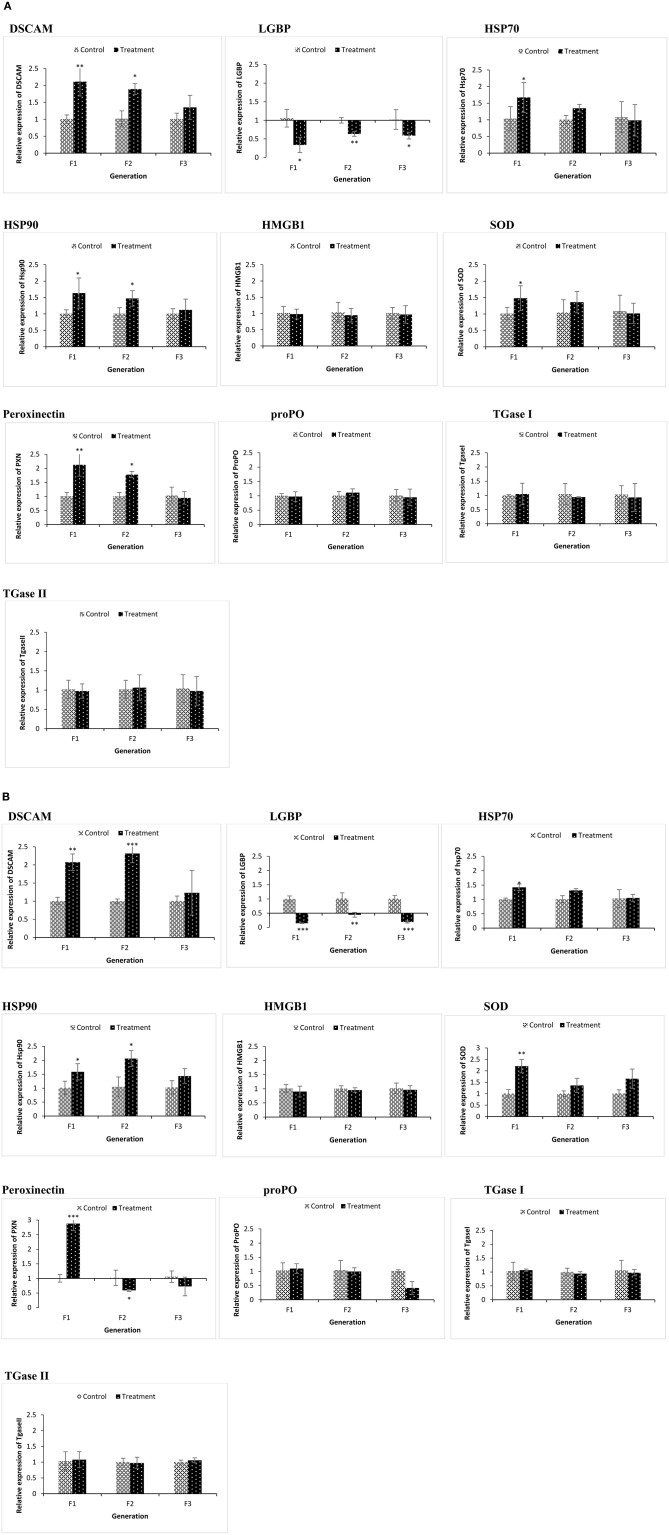
During the common garden experiment, age and size synchronized one-day old larvae originating from phloroglucinol treated and non-treated parents were used. To assess the immune gene expression profile of non-infected animals, transcription of immune-related genes in F1, F2, and F3 larvae **(A)** and F1, F2, and F3 juveniles **(B)** was studied. Error bars represent the standard deviation (*n* = 3) and significant differences between the treatment and control groups at each respective generation are indicated by *p < 0.05, **p < 0.01, ***P < 0.001.

**Figure 5:**
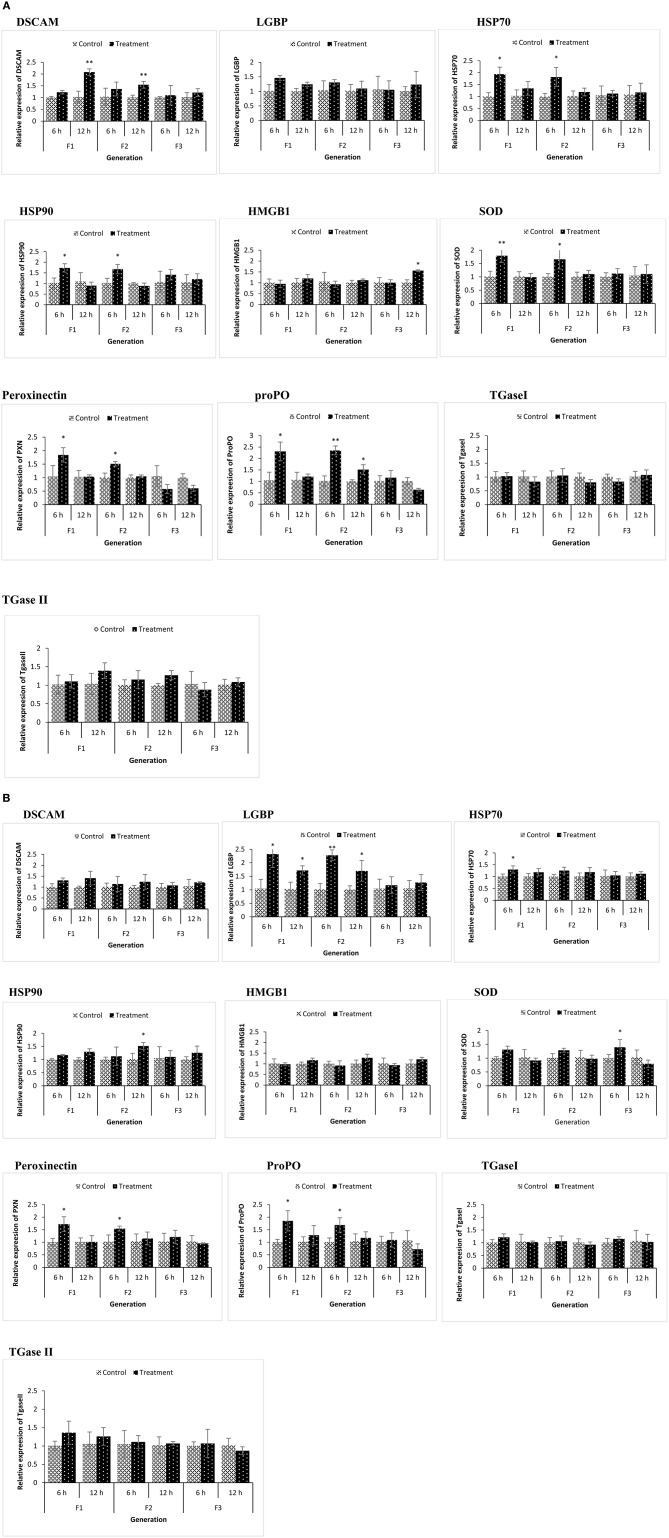
During the common garden experiment, age and size synchronized 1-day old larvae originating from phloroglucinol treated and non-treated parents were infected with *Vibrio parahaemolyticus* (AHPND strain M0904) or *Vibrio harveyi* (strain BB120). To assess the immune gene expression profile of infected animals originating from treated or non-treated parents, transcription of immune-related genes in F1, F2, and F3 larvae infected with *Vibrio parahaemolyticus* **(A)** or *Vibrio harveyi* **(B)** was studied at 6 and 12 h post infection. Error bars represent the standard deviation (*n* = 3) and significant differences between the treatment and control groups at each respective generation are indicated by *p < 0.05, **p < 0.01.

We examined if resistance against biotic (*Vibrio* spp. infections) or abiotic (lethal heat shock) stressor in the axenic nauplii collected from TF1 to TF3 generations was associated with altered immune-related gene expression during the common garden experiment. Newly hatched larvae from both control and treatment groups of F1–F3 generations were sampled. Remaining animals from both control and treatment groups (F1–F3) were cultured under conventional control condition without any challenge or treatment and were sampled at the juvenile (16 days old) life stage (Figures 4A,B). To evaluate the response to the infection and to understand the possible transgenerational immune priming effects of the compound, the same innate immune-related genes were examined in F1 to F3 larvae at 6 and 12 h post infection (Figures 5A,B). This result also provided some information on the possible differences in reaction of brine shrimp innate immunity against different pathogens in the presence and absence of the compound-induced immune priming.

In the phloroglucinol-treated, non-challenged TF1 to TF2 larvae and juveniles, mRNA levels for the pattern recognition receptor down syndrome cell adhesion molecule (*DSCAM*) were significantly upregulated (2.07- and 2.31-fold rise in TF1 and TF2 juveniles and 2.11- and 1.89-fold rise in TF1 and TF2 larvae) as compared to animals of CF1 to CF2, whereas mRNA levels for the lipopolysaccharide and beta-1,3-glucan binding protein (*LGBP*) were significantly downregulated (~0.6 fold decline in juveniles and ~ 0.1 fold in larvae) (Figures 4A,B). Transcript levels for the peroxinectin gene were significantly upregulated in TF1 (2.12-fold rise) and TF2 larvae (~1.8-fold rise). However, in juveniles, *peroxinectin* mRNA levels were only significantly upregulated in TF1 (2.9-fold rise) and downregulated in TF2 (0.59-fold decline) and TF3 (0.72-fold decline) animals. Superoxide dismutase (*SOD*) mRNA levels were significantly upregulated in TF1 juveniles (2.2-fold rise) and Heat shock protein 90 (*HSP90*) mRNA levels were significantly upregulated in TF1 (1.58-fold rise) and TF2 (2-fold rise) juveniles, but in larvae, the relative expression was <1.5, whereas *HSP70* was significantly upregulated (1.67-fold rise) only in the TF1 larvae. Moreover, there were no significant changes in the expression of *HMGB1, ProPO, TGase I*, and *TGase II* either in TF1 to TF3 larvae or juveniles. These results of “phloroglucinol treated, non-challenged” gene expression profile suggest that immune receptor *DSCAM, LGBP*, and *peroxinectin* were differentially induced in TF1 to TF3 generation of brine shrimp.

Some immune-related genes of phloroglucinol-treated and challenged (AHPND strain *V. parahaemolyticus* or *V. harveyi*) brine shrimp larvae were upregulated in a time-dependent manner (Figures 5A,B). *DSCAM* was significantly upregulated in TF1 and TF2 larvae challenged with *V. parahaemolyticus*, and *LGBP* was significantly upregulated in TF1 and TF2 larvae challenged with *V. harveyi*. Regardless of the vibrio species used, the expression of *ProPO* and *PXN* was significantly upregulated in TF1 and TF2 larvae at 6 h post infection*. SOD, HSP70*, and *HSP90* only showed significantly elevated expression levels in TF1–TF2 larvae challenged with *V. parahaemolyticus*. These results indicate that *Vibrio* exposure had a stimulatory effect and significantly modulates the expression of innate immune-related genes in TF1–TF3 brine shrimp animals.

**Figure 6:**
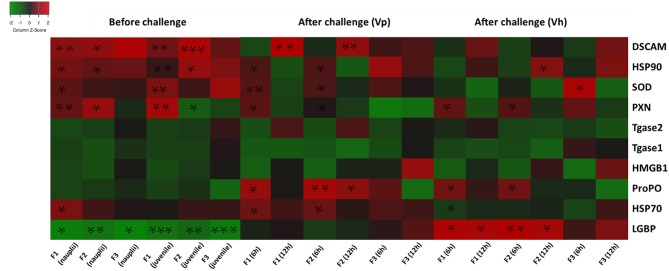
Heatmap showing transcription levels for innate immune-related genes analyzed in three subsequent brine shrimp generations F1, F2, and F3 originating from phloroglucinol-treated parents. Results for non-infected larvae and juveniles and for larvae infected with *V. parahaemolyticus* or *V. harveyi* are presented. Gene upregulation in TF1 to TF3 as compared to CF1 to CF3 is visualized in red and gene downregulation upregulation in TF1 to TF3 as compared to CF1 to CF3 in green. An asterisk * represents a significant difference between the treatment and control groups at each respective generation (*P < 0.05, **P < 0.01, ***P < 0.001).

The results are also summarized in a heatmap (Figure 6). Overall, the results showed that the transcription profile of innate immune-related genes was differentially expressed in TF1 to TF3 brine shrimp animals, and it further supports the idea that phloroglucinol might be involved in inducing transgenerational-inherited robust phenotype in brine shrimps.

### Global Changes in DNA Methylation (5-mC%) in F1–F3 Brine Shrimp Whose Parental Generation (F0) Was Treated With Phloroglucinol

**Figure 7:**
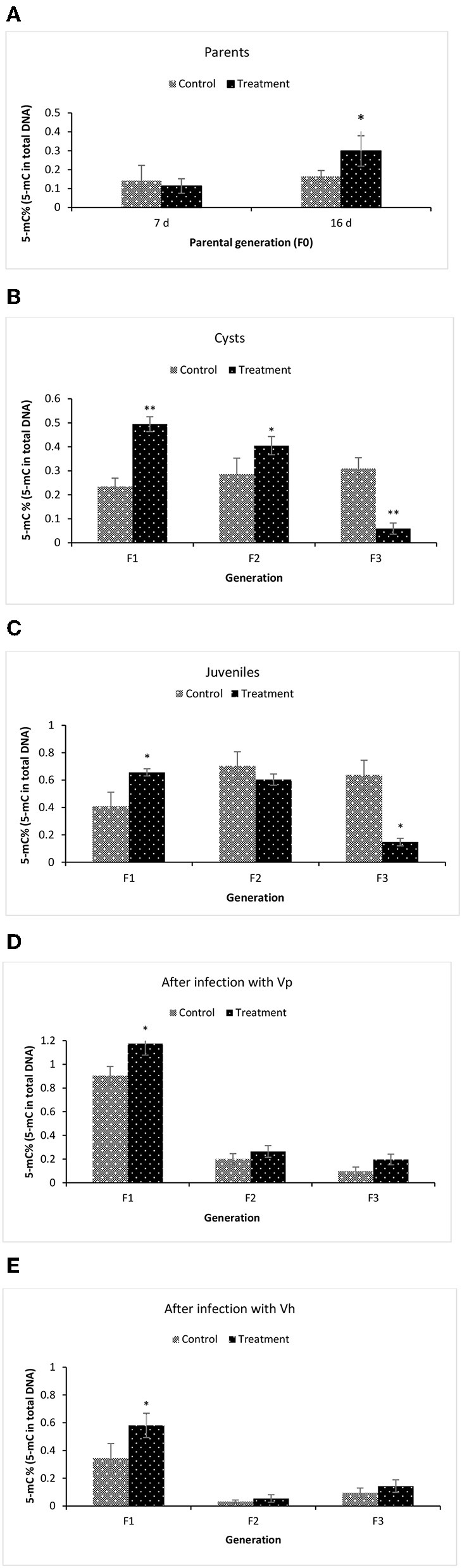
Global changes in DNA methylation (5-mC%) in F1–F3 brine shrimp whose parental generation (F0) was treated with phloroglucinol. Global methylated DNA levels (5-mC % to total DNA) were analyzed from brine shrimp parents **(A)** and three successive F1, F2, and F3 generations each time from non-infected cysts **(B)**, non-infected juveniles **(C)**, *V. parahaemolyticus-*infected larvae at 12 h post challenge **(D)** and *V. harveyi*-infected larvae at 12 h post challenge **(E)**. The MethylFlash Global DNA Methylation (5-mC) ELISA Kit (Epigentek, USA) was used. Error bars represent the standard deviation (*n* = 3) and significant difference (*P* < 0.05) in DNA methylation levels between the control and treatment groups are indicated with an asterisk (^*^, **P < 0.01).

To examine the underlying mechanism of phloroglucinol-induced transgenerational-inherited increased resistance against bacterial challenge, we determined global DNA methylation levels (5-mC % to total DNA) in the parental generation and subsequent generation (TF1–TF3) animals. The global DNA methylation level was measured in (i) the parental generations TF0 and CF0 both at DAH7 and at DAH16, (ii) TF1 to TF3 and CF1 to CF3 cysts, (iii) TF1 to TF3 and CF1 to CF3 juveniles, and (iv) larvae 12 h post challenge (*V. parahaemolyticus* or *V. harveyi*) (Figure 7). At DAH16, the DNA methylation level in the treated parental generation (TF0) was significantly higher than the non-treated parental controls (CF0) (Figure 7A). Interestingly, a significant higher DNA methylation level (5-mC%) was observed in TF1 and TF2 brine shrimp cysts as compared to their respective controls, whereas this was no longer the case in the TF3 brine shrimp (Figure 7B). Further, we investigated whether global DNA methylation found in the dormant embryos (cysts) was also present in juveniles and if methylation was related to pathogen exposure. Results showed that the DNA methylation level in the juveniles was significantly higher in TF1 as compared to CF1 (Figure 7C). DNA methylation in the brine shrimp larvae at 12 h post exposure with *V. parahaemolyticus* or *V. harveyi* showed a similar pattern, revealing a significant difference (increase) in 5-mC% for TF1 animals compared to their respective controls. The 5-mC% was statistically the same between TF2 and CF2 and between TF3 and CF3 (Figures 7D,E). DNA methylation levels following phloroglucinol treatment might play a role in the inheritance of the bacteria-resistant phenotype. However, regardless of whether DNA levels were increased or decreased, TF1 to TF3 all had higher vibrio resistance properties. Thus, we could not find a link between DNA methylation levels and higher disease resistance.

### Global Changes of m6A (N6-methyladenosine) RNA Methylation in F1–F3 Brine Shrimp Whose Parental Generation (F0) Was Treated With Phloroglucinol

**Figure 8:**
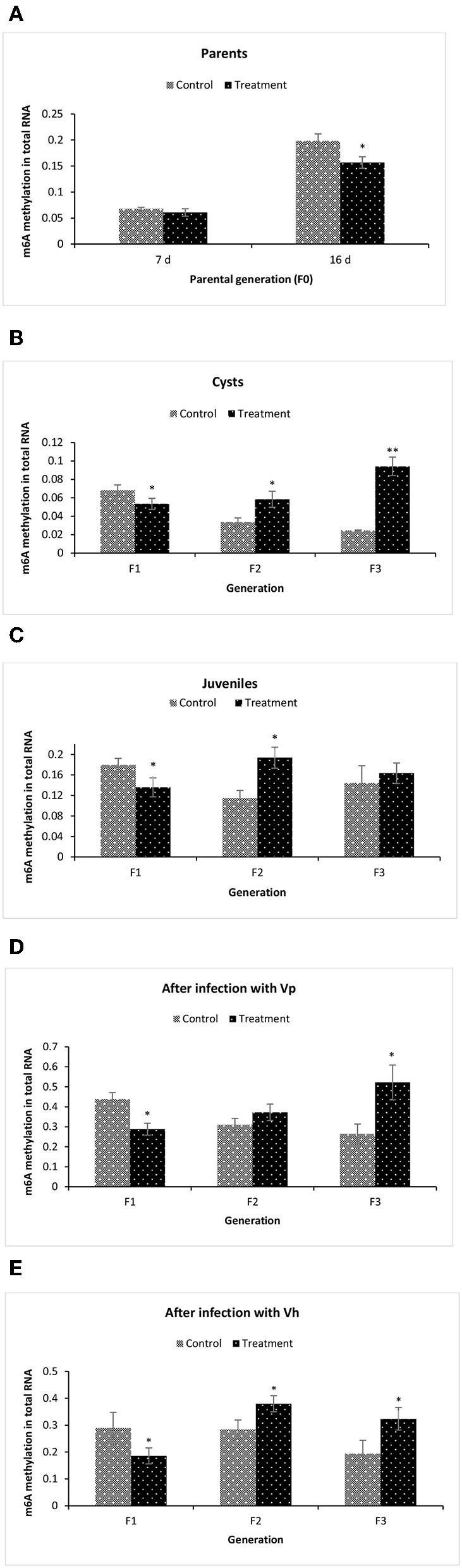
Global changes of m6A (N6-methyladenosine) RNA methylation in F1–F3 brine shrimp whose parental generation (F0) was treated with phloroglucinol. Total RNA was extracted and N6-methyladenosine (m6A) RNA methylation was analyzed from brine shrimp parents **(A)** and three successive F1, F2, and F3 generations each time from non-infected cysts **(B)**, non-infected juveniles **(C)**, *V. parahaemolyticus-*infected larvae at 12 h post challenge **(D)** and *V. harveyi*-infected larvae at 12 h post challenge **(E)**. For this purpose, the EpiQuik™ m6A RNA Methylation Quantification Kit was used. Error bars represent the standard deviation (*n* = 3) and significant difference (*P* < 0.05) in RNA methylation levels between the control and treatment groups are indicated with an asterisk (^*, **^P < 0.01).

To gain more insights related to phenotypic observation of phloroglucinol-induced transgenerational inherited increased resistance, next we aimed to identify the m6A (N6-methyladenosine) RNA methylation level in the brine shrimp. The m6A RNA methylation level was measured in (i) the parental generations TF0 and CF0 both at DAH7 and at DAH16, (ii) TF1 to TF3 and CF1 to CF3 cysts, (iii) TF1 to TF3 and CF1 to CF3 juveniles, and in (iv) larvae 12 h post challenge (*V. parahaemolyticus* or *V. harveyi*) (Figure 8). At DAH7, m6A RNA methylation levels were statistically the same for TF0 and CF0. However, on DAH16, the RNA of TF0 showed a significantly lower m6A methylation level as compared to that of CF0 (Figure 8A). The m6A RNA methylation level was significantly lower in TF1 cysts as compared to CF1. On the other hand, the m6A RNA methylation levels were significantly higher in TF2 and TF3 cysts as compared to their respective controls CF2 and CF3 (Figure 8B). In TF1 juveniles, the m6A RNA methylation level was (i) significantly lower in TF1 as compared to CF1, (ii) significantly higher in TF2 juveniles as compared to CF2 juveniles, and (iii) statistically the same for TF3 and CF3 juveniles (Figure 8C). The m6A RNA methylation level in brine shrimp larvae 12 h post infection with *V. parahaemolyticus* or *V. harveyi* revealed the following: (i) a significantly lower m6A RNA methylation level in TF1 larvae compared to CF1, and (ii) a significant increase in m6A RNA methylation level in TF2 and TF3 larvae as compared to their respective controls (Figures 8D,E). RNA (m6A) methylation levels following phloroglucinol treatment might play a role in the inheritance of the bacteria-resistant phenotype. However, regardless of whether RNA (m6A) methylation levels were increased or decreased, TF1 to TF3 all had higher *Vibrio* resistance properties. Thus, we could not find a link between RNA (m6A) methylation levels and higher disease resistance.

### Acetylation of Total Histone H3 and H4 Followed by Modulation of Multiple H3 and H4 Epigenetic Modifications in F1–F3 Brine Shrimp Whose Parental Generation (TF0) Was Treated With Phloroglucinol

We further studied the histone modifications (global and specific) to test whether histone modifications have also any role in phloroglucinol-induced observed phenotype of transgenerational inherited increased resistance in brine shrimp.

**Figure 9:**
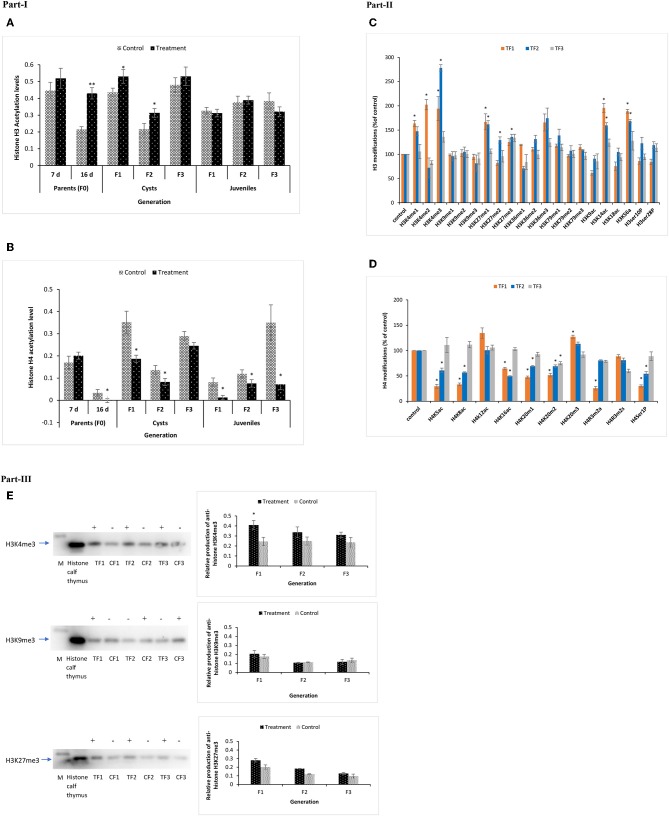
Total acetylation of histone H3 and H4 and multiple modifications of H3 and H4 in F1 to F3 brine shrimp whose parental generation (TF0) was treated with phloroglucinol or non-treated (CF0). Total histone was extracted from the control and treatments groups (in three biological replicates) of F0 (DAH7 and DAH16) and F1, F2, and F3 generations (cysts and juveniles) by the direct acid extraction method. Part I. Alteration of total H3 **(A)** and H4 **(B)** histone acetylation patterns in the brine shrimp parental generation TF0 and three successive generations of brine shrimp cysts and juveniles using a fluorometric kit (Abcam). Statistical data are represented as the mean of three biological replicates, error bars represent the standard deviation (*n* = 3), and significant differences (*P* < 0.05) between control and treatment groups are indicated with an asterisk (*). Part II. Multiple histone H3 and H4 modified proteins in F1–F3 brine shrimp cysts. **(C)** H3 Histone 21 post-translational modification states were analyzed from cysts of treatment and control groups. Results showed significantly altered histone modifications in TF1, TF2, and TF3 generations as compared to the respective controls. **(D)** H4 Histone 10 post-translational modifications were analyzed in cysts of both the treatment and control group and results showed significantly altered histone modifications in TF1, TF2, and TF3 generations as compared to the respective controls. Statistical data are presented as the standard deviation (*n* = 2), and significant differences (*P* < 0.05) between control and treatment groups are indicated with an asterisk (*). Part III. Elevated H3K4me3 and H3K27me3 levels and decreased H3K9me3 levels in TF1–TF3 brine shrimp were verified by Western blotting. Western blotting was performed for H3K4me3, H3K27me3, and H3K9me3 in histone extracts from cysts of three subsequent generations from both the treatment and control groups. The representative cropped blot shown here highlights the protein of interest (full length blots are included in the Figure S6). “+” represents higher protein expression level and “–” represents lower protein expression level relative to histone calf thymus (positive control), which was regarded as 1.0. Graphical representation and quantitative analysis of optical density of histone H3K4me3, H3K27me3, and H3K4me3 levels in brine shrimp were performed by Image software. Error bars represent the standard deviation (*n* = 3). Significant differences (*P* < 0.05) as compared to the positive control (*P* < 0.05) (*) are indicated with an asterisk (*).

At DAH7, total histone H3 acetylation levels in TF0 parents and CF0 controls were statistically the same. However, at DAH16, the total histone H3 acetylation level was significantly higher in phloroglucinol-treated TF0 parents as compared to CF0 controls. Total histone H3 acetylation was significantly higher in the TF1 to TF2 cysts vs. their respective controls. For juveniles, the total histone H3 acetylation levels in treated brine shrimp and controls were always statistically the same (Figure 9A) since environmental condition or brine shrimp after certain life stage might have influenced the pattern of acetylation and are not that evident and could not hold significant changes in juveniles. In regard to total histone H4 acetylation, phloroglucinol treatment leads to significantly lower total histone H4 acetylation levels in TF0 parents, in TF1 to TF2 cysts, and in TF1 to TF3 juveniles as compared to their respective controls (Figure 9B).

Next, to gain insight into specific histone modifications, the occurrence of 21 histone H3 protein modifications (15 different lysine methylations, 4 lysine acetylations, and 2 serine phosphorylations) and 10 histone H4 protein modifications (3 different lysine methylations, 2 arginine methylations, 4 lysine acetylations, and 1 serine phosphorylation) was examined for the TF1 to TF3 and CF1 to CF3 cysts. Increased methylation was observed for H3K4 and H3K27 and increased acetylation for H3K14 and H3K56. Specifically, the H3K4me1, H3K4me2, H3K4me3, H3K27me1, H3K14ac, and H3K56ac contents were significantly higher for TF1 vs. CF1 cysts. For TF2 vs. CF2 cysts, H3K4me3, H3K27me1, H3K27me2, H3K27me3, H3K14ac, and H3K56ac were significantly higher. Data for TF3 vs. CF3 were statistically the same (Figure 9C). In case of histone H4 protein modifications, H4K20m2 was significantly lower in TF1–TF3 cysts; H4K5ac, H4K8ac, H4K16ac, H4K20m1, and H4Ser1P contents were significantly lower in the TF1 to TF2 cysts while H4K20m3 was significantly lower in TF1 cysts. Moreover, the H4R3m2s and H4K12ac contents were statistically the same (Figure 9D).

To verify the reliability of the data obtained by the multiplex histone ELISA, H3K4me3, H3K27me3, and H3K9me3 contents for TF1–TF3 and CF1–CF3 cysts were also examined by Western blotting. ELISA results for H3K4me3, H3K27me3, and H3K9me3 were successfully confirmed by Western blotting (Figure 9E and Figure S6). Overall, our data suggest that there were significant epigenetic modifications on histone H3 tails, including several euchromatin marks (i.e., global acetylation of H3, H3K4me3, H3K4me1, H3K4me2, H3K14ac, and H3K56ac) and heterochromatin marks (H3K27me1 and H3K27me3) in the TF1–TF3 brine shrimp cysts. These results indicate that phloroglucinol treatment in the parental generation might cause epigenetic modifications of histone H3 that can be inherited by three subsequent generations of untreated animals. These modifications may be in part responsible for observed increased robustness in the larvae of TF1–TF3 generation compared to the respective controls.

## Discussion

At present, there is increasing evidence for epigenetic inheritance of acquired phenotypic traits across multiple generations (18, 20, 41–44). Some of these phenotypes were developed in response to changing environment (13). Mechanisms by which an organism acquires phenotypes and passes them to the subsequent generations through germline is still of major interest. A link has been established in a previous study between increased levels of HSP70 production, induced by daily non-lethal heat shocks in parental generation, and epigenetically regulated transgenerational inheritance of increased biotic and abiotic resistance phenotypes in clonal parthenogenetic *Artemia* (18). The aim of the current study was to determine the possibility for replacement of costly and labor-intensive heat shock approach with the application of HSP inducing plant-based compound in shrimps. This could be an innovative method for disease prevention and control measures in shrimp farming industry.

In the present study, by using brine shrimp (*A. franciscana*) as model organism, we showed that frequent application of phloroglucinol at early life induces phenotypic traits (i.e., tolerance against lethal heat shock and vibriosis caused by pathogenic *Vibrio parahaemolyticus* AHPND strain as well as *V. harveyi*) at the end of the monitoring period, and these acquired traits can be transmitted to three successive, unexposed generations (Figure 3 and Figure S3). To evaluate animal disease resistance across F1, F2, and F3 generations, the survival tests were carried under both conventional (germ-associated ovoviviparously produced nauplii at each generation) and common garden gnotobiotic (axenic system in which the known bacteria are manually added) conditions. Gnotobiotic common garden experimental systems exclude the possibility of confounding factors, such as differential microbial community and different size/age of larvae, e.g., across generations (18). Under both these experimental conditions, we observed similar phenotypes of acquired resistance against abiotic and biotic stressors in *Artemia*. This indicates that information from the treated parents was transmitted to the progenies. These observed phenotypes were associated with several innate immune-related mRNA expressions and epigenetic marks.

In transgenerational inheritance, the epigenetic information can be transferred through several mechanisms such as chromatin modifications (DNA methylation, histone modifications), non-coding RNA (ncRNA) molecules, and RNA methylation. These acquired epigenetic marks can be stably passed through the gametes and persist for multiple generations (9, 45). The observed phloroglucinol-induced transgenerational inheritance of robustness could be a result of various molecular events. To understand the possible mechanism behind transgenerational inherited robust phenotypes, we first analyzed the expression of innate immune-related genes in brine shrimp across generations (TF1–TF3) and in two life stages (larvae and juveniles). Moreover, the invertebrate defense system depends on both constitutive and inducible immune mechanisms, and phloroglucinol-induced immune gene expression at both constitutive level and after bacteria challenge might possibly facilitate resistance to bacterial infection and heat stress (46, 47). Therefore, in the present study, both possibilities were explored.

Our differential gene expression results suggested that the observed phenomenon could be associated, at least in part, with the induction of the pattern recognition receptors (PRRs), e.g., *DSCAM* and *LGBP*. The pathogen recognition receptors (PRRs), e.g., *DSCAM* and *LGBP*, are germline-encoded host sensors of the innate immune system that detect pathogen-associated molecular patterns (PAMPs) (48). *DSCAM* is a complex organized hypervariable protein and the molecular diversity of *DSCAM* plays an important role in the specificity derived through alternative splicing mechanisms (49–51). More advanced studies during the past decade have demonstrated that *DSCAM* is the most likely candidate for innate immune specificity and adaptive-like innate immune memory in invertebrates, including crustaceans, since *DSCAM* might be involved in pathogen-specific splice variants after immune challenge and microbial neutralizing after vaccination (49, 52–60). *LGBP* regulates innate immune defense in invertebrates against Gram-negative bacteria (61, 62). *LGBP* is also involved in protection of shrimp against infections with Gram-positive bacteria and fungi (63, 64). In our study, *DSCAM* mRNA levels were significantly elevated in phloroglucinol treated, non-challenged brine shrimp but whose ancestors were phloroglucinol treated (TF1–TF3) in both larvae and juveniles and in post-*Vibrio* exposed larvae (Figures 4, 5). *LGBP* expression was only upregulated after *Vibrio* challenge. Surprisingly, *DSCAM* was more responsive to *V. parahaemolyticus* and *LGBP* to *V. harveyi*. Results showed that these PRR could be involved in the phloroglucinol-induced transgenerational robustness and brine shrimp PRR could discriminate to some extent between the two *Vibrio* strains.

Along with PRRs, the expression of other important immune-related genes was studied in three subsequent brine shrimp generations, namely, genes encoding for signal molecules (*HSP70, HSP90*, and *HMGB1*), genes responsible for humoral immune defense (*proPO, peroxinectin, TGase*), and the antioxidant defense gene *SOD*. Our results suggested that the observed phenomenon could also be associated, at least in part, with the induction of the signaling molecules *HSP70* and *HSP90*. A positive correlation between elevated *HSP70* and *HSP90* transcript levels and increased resistance phenotypes was observed in the TF1 and TF2 generation progenies (whose ancestors were treated with the compound) before challenge and in the TF1–TF3 generations after being challenged with the two different strains of *Vibrio* (i.e., *V. parahaemolyticus* and *V. harveyi*). This result is consistent with the known important roles of HSP70 and HSP90 signaling proteins in defining the resistance of organisms against stressor by performing multifaceted functions, such as acting as molecular chaperone for proteins, functioning as danger-associated molecular pattern (DAMP) during inflammation and various cellular processes (65, 66) and/or participating in the activation of cell surface innate immune receptors, thereby modulating many aspects of host's immune responses (21). Additionally, based on our results, it appears that *HSP90* mRNA expression plays a bigger role in the transgenerational inherited resistance induced by phloroglucinol compared to *HSP70*. We found that in the absence of any stressors, *HSP90* mRNA stayed significantly upregulated in the untreated progenies of the parents that went through compound treatment, at TF1 and TF2 generations, during both nauplii and juvenile stages. However, after a challenge with *V. parahaemolyticus* or *V. harveyi*, it appears that both *HSP70* and *HSP90* mRNA followed similar expression patterns at different time points and different generations. Increased levels of *HSP70* may not be the only factor for transgenerational increased resistance of the animals against pathogenic challenge, and other molecular chaperones, such as *HSP40, HSP60*, and *HSP90*, may play a bigger role (67, 68). Our results suggest that application of phloroglucinol has an effect on keeping signaling molecules and more importantly *HSP70* and *HSP90* mRNA primed for a more efficient response during the stressful condition (21, 69). In our study, the increased levels of *HSP90* gene expression under the control conditions where the animals were not challenged were followed by increased mRNA levels of innate immune-related genes including *DSCAM, PXN*, and *SOD* in the same experimental conditions and animals' life stage.

Among the immunity-related genes, *proPO* is an important constituent of the proPO cascade reaction that plays an important role in invertebrate protection against invading pathogens by melanization of pathogens (70, 71). *Peroxinectin* (PXN) is another defense-related gene and is a cell adhesive protein known to be strongly associated with the proPO system (72). It regulates the expression of antimicrobial peptides (AMPs) in invertebrates (73, 74). The defense molecule *TGase* is another important constituent of the innate immune repertoire responsible for catalyzing the clotting reaction (75). Based on our results, the genes encoding for the immune effectors *proPO* and *TGase* (I and II) mRNA were not upregulated in TF1–TF3 generation (both nauplii and juvenile) in the absence of challenge. However, despite the absence of any significant change in the gene expression levels of *proPO* or *TGase* in these animals, a significant upregulation of *PXN* was observed at TF1 (both nauplii and juvenile) and TF2 generations (only nauplii stage). Surprisingly, *PXN* was downregulated at TF2 (juvenile) and TF3 (both nauplii and juveniles) generations. From this result, it also appears that compound application in parental generation induces the production of *PXN*. This priming effect is passed on to the first two subsequent generations, but in the absence of the compound, they fade away and are reversed. However, once nauplii were challenged with either *V. parahaemolyticus* or *V. harveyi*, an increase in levels of *proPO* mRNA expression was observed across TF1–TF3 generations. Interestingly, the *proPO* gene expression patterns were followed by *PXN* once the animals were challenged with either of the pathogens. These results show a strong link between the two genes. By comparing the gene expression analysis results of *proPO* and *PXN* before and during the challenge, the only possible conclusion is that the compound application in the parental generation induces some kind of modifications on these immune-related genes that could result in the faster and more efficient expression of the mRNA during the challenge. Significantly elevated *proPO* expression levels in treated shrimp were correlated with higher survival rates in subsequent generations. However, we did not measure the phenol oxidase enzyme activity in shrimp, nor did we analyze protein expression at that time. However, we did collect samples for a future LC-MS/MS study to identify differentially expressed and regulated proteins. Free radicals and reactive oxygen species (ROS) are produced in the metabolic pathways of aerobic cells. The activated innate immune system also engages in phagocytosis to eliminate ROS and invading microorganisms with the aid of the antioxidant enzymes such as SOD (76). In shrimp, SOD plays an important role in protection against *V. parahaemolyticus* and WSSV infection (16, 76). In the present study, a significant increase in levels of *SOD* mRNA was recorded, only at TF1 generation, in unchallenged nauplii and juveniles. After the challenge, *SOD* gene showed a *Vibrio*-specific expression. The gene was upregulated during both challenges at 6 hpi. However, the difference between control and treatment was significant only at TF1 and TF2 in the group that was challenged with *V. parahaemolyticus*. After challenge with *V. harveyi*, a significant upregulation of *SOD* gene was observed only at TF3 generation during the first 6 hpi compared to the respective control. However, despite this increase, the mRNA level was slightly reduced at TF1–TF3 generations at 12 hpi compared to the respective controls. This was not the case once the animals were challenged with *V. parahaemolyticus*. These results suggest that treating the parental generation with phloroglucinol compound increases animals' superoxidase dismutase activity in subsequent generations, but the induced effect is reduced during later generations. In a study, it has been observed that a heat shock protein-inducing product mediates its HSP70-inducing effect in the brine shrimp larvae by initial generation of ROS, such as superoxide anion and H_2_O_2_ against pathogenic *Vibrio* and there is a positive correlation between H_2_O_2_ release and HSP70 production (24). Similarly, it is shown that phloroglucinol application can induce production of free radicals in animals' cell (77).

The result of our gene expression analysis indicated that the compound application in parental generation could transgenerationally prime the innate immune system of the three subsequent non-treated generations. Additionally, from our results, it appears that the animal's innate immunity could discriminate to some extent between the two *Vibrio* strains and mount an individual innate immune response against each species.

Since some of these innate immune genes stayed primed or upregulated in the absence of environmental stressors, we next aimed at understanding the possible mechanisms behind this transgenerational inheritance. Recent studies provided evidence for epigenetic mechanisms such as chromatin modifications, DNA methylation, histone modifications, long-lived non-coding RNA, and, very recently, RNA methylation in transgenerational reprogramming across generations (78, 79). The occurrence of epigenetic modifications results in chromatin remodeling, which, in turn, affects the transcription status of innate immune genes (20). In transgenerational inheritance, the epigenetic information can be transferred through several mechanisms such as DNA methylation, RNA methylation, and histone modifications. These acquired epigenetic marks can be stably passed through the gametes and persist for multiple generations (9, 45). The observed phloroglucinol-induced transgenerational inheritance of robustness and elevated innate immune gene expressions could be a result of some epigenetic modifications.

At first, we measured DNA methylation in the brine shrimp, since it is the most widely studied and prevalent epigenetic mark, and typically involves addition of methyl groups primarily at the cytosine base when it is adjacent to guanine base to produce 5-methylcytidine (5-mC). Moreover, DNA methylation changes the way DNA binds and interacts with proteins to regulate the transcription, and recent reports also suggested that methylation was involved in defining exon recognition or exon–intron boundaries that affects splice variant production (80, 81). In the present study, a significant increase in global DNA methylation was observed in treated brine shrimp TF0 parents (DAH16) and in subsequent generations of cysts (TF1–TF2), juveniles, and *Vibrio* challenged larvae (TF1). The results highlight possible involvement of DNA methylation in the epigenetic modifications for creating a resistant phenotype and gene expression regulation in transgenerational brine shrimp. Global DNA methylation can be further varied depending on the genotype, different environmental stressors, and their interactions (82). Here, we observed lower levels of global DNA methylation under phloroglucinol-induced but no pathogen exposure (parents, cysts, and juveniles) as compared to phloroglucinol-treated and pathogen-exposed larvae (AHPND strain *V. parahaemolyticus*), indicating that both phloroglucinol and pathogen might have impacted DNA methylation in brine shrimp. There are few reports showing that DNA methylation is affected by environmental stress such as heat shock in the red flour beetle *Tribolium castaneum* (83) and across generation in parthenogenetic *A. franciscana* (18). Likewise, the contaminated water environment and chronic gamma irradiation have been reported to modulate the DNA methylation level across generations in *Daphnia* (84, 85). Since DNA methylation was not uniform across generation, we could not provide a true causal link between DNA methylation and induced inherited bacteria-resistant phenotype. However, this does not completely rule out the possibility that DNA methylation might possibly be involved in developing resistance in brine shrimp after phloroglucinol treatment to the parental generation. It needs to be stated that there are also some discrepancies in the reports on the level of global DNA methylation level as analyzed with different methods such as antibody-based or MS-based methods (84, 86). Here global DNA methylation was only analyzed by an antibody-based method. In the future, it is advisable to study DNA methylation in *Artemia* on a finer scale targeting specific genes using more sensitive methodologies such as bisulphite sequencing.

Recent studies strongly suggest that RNAs also have dynamic regulatory roles in several biological processes, analogous to epigenetic DNA and histone modifications (87, 88). RNA methylation adds a new dimension in regulating post-transcriptional gene expression, which could affect various aspects of RNA metabolism, e.g., mRNA translation, which can directly impact protein production. N6-methyladenosine (m6A), which refers to methylation of adenosine base at the nitrogen-6 position, is considered the most abundant and conserved internal modification in mRNA and long non-coding RNA (89, 90). It plays an important role in multiple levels of regulation such as splicing, translation, degradation, and possibly microRNA regulated fine-tuning of gene expression (91–94). Therefore, it was interesting to investigate if m6A RNA methylation was associated with the observed transgenerational effects in our brine shrimp model system. There was a significant decrease in global m6A RNA methylation in TF0 parents and TF1 brine shrimp. However, global m6A RNA methylation significantly increased in TF2 and TF3 generations cysts, juveniles (non-challenged), and *Vibrio* challenged larvae (Figure 8). Castro-Vargas et al. (88) used the invertebrate beetle *Tenebrio molitor* model system and reported differential global RNA methylation having a role within generations' immune priming phenomena rather than DNA methylation. In the present study, a differential RNA methylation was observed across generations, a lower percentage of m6A RNA methylation was observed in the parents and TF1, whereas a higher percentage of m6A RNA methylation was observed in TF2–TF3 generation brine shrimp. However, the exact role is not clear from the current pattern across generations and we could not provide a true causal link between RNA (m6A) methylation and the induced inherited bacterial resistant phenotype. Nevertheless, this does not completely rule out the possibility that m6A RNA methylations might have possibly been involved in developing resistance in brine shrimp after phloroglucinol treatment to the parental generation and in transgenerational inherited robustness in brine shrimp.

Histone tail modifications are examples of epigenetic mechanisms. Indeed, such modifications in the promoters of defense genes have been shown to correlate with transgenerational-induced resistance against abiotic (95, 96) and biotic stresses (97) in the Arabidopsis plant model. In the present study, we observed alterations in the global acetylation of H3 and H4 as well as in acetylation/methylation of specific H3 and H4 lysin tails. Our results indicate that perhaps phloroglucinol-induced transgenerational inheritance of robustness was achieved by histone epigenetic molecular changes (75, 76). Histone acetylation is one of the most studied modifications as facilitated by histone acetyltransferases (HATs), and it can be reversed also by histone deacetylases (98, 99). Generally, histone acetylations (specific or global) are reported to be linked with euchromatin formation and increased mRNA expression. Histone acetylations such as H3K14ac and H3K9ac at the promoter regions are associated with transcriptional activation and promoting gene expression. Elevated total H3 histone acetylation has been reported in successive generations upon environmental heat stress in *Artemia* (18). Interestingly, in the current study, a significant increase in global histone H3 acetylation was observed in the parents (DAH16) and subsequent generations of brine shrimp cysts (TF1–TF2). At TF3 generation a slight histone hyperacetylation was observed, but it was not significantly different from CF3. Based on this result, it was apparent that in the absence of compound treatment, histone hyperacetylation was lost over generations. But in the juveniles, there was no significant change in global histone H3 acetylation. This may be due to the fact that brine shrimp after a certain life stage or environmental culture condition may influence the pattern of histone acetylation and therefore are not that evident and could not be detected in juveniles. Along with the transgenerational hyperacetylation global H3, a significant increase in levels of H3K14ac and H3K56ac was observed in the cysts belonging to TF1 and TF2 generations. In contrast, in case of H4, the global acetylation was significantly reduced in both the parental generation DAH16 and the TF1–TF3 cysts and juveniles. Additionally, we also tested the more specific lysine modification on H4. We found that H4K5, H4K8, H4K12, and H4K16 were also hypoacetylated in the cysts from F1 and F2 generations (Figures 9A,B). However, at F3 generation, the difference between treatment and control groups was not significant anymore. So, these results indicate that histone acetylation indeed does play a role in phloroglucinol-induced transgenerational-resistant phenotype in brine shrimp.

Histone methylation is another major modification for epigenetic gene regulation that is associated with either transcriptional activation or gene silencing depending on the sites/positions and degree of methylation (100). In general, histone H3K4 methylations mono-, di-, or trimethylation are mostly associated with gene activation. In particular, trimethylation H3K4me3 is considered as a hallmark for gene activation and directly involved in transcriptional activation (101, 102). Genome-wide distribution patterns of H3K4 methylations revealed that all three types of methylation, H3K4me1, H3K4me2, and H3K4me3, are exclusively present in the genes and promoter regions. H3K4me3 and H3K4me2 are mostly enriched in the promoters and particularly in the 5′ end of transcribed regions with H3K4me3 slightly upstream of H3K4me2 and H3K4me1 depleted in promoters but enriched within transcribed regions, which suggests that H3K4me3 and H3K4me2 might be involved in the transcription initiation and early elongation, whereas H3K4me1 was primarily involved during transcription elongation (103). Interestingly, in the current study, a significant increase in H3K4me3 has been observed in the cysts from subsequent generations (TF1–TF3) of brine shrimp. Along with this trimethylation H3K4me3, a significant increase in two other methylated modified proteins at H3K4 sites, i.e., H3K4me1 in TF1–TF2 generations and H3K4me2 in TF1 generation cysts, was observed (Figures 9C,D). Based on this result, it was evident that a significant increase of methylation has been observed in H3K4 sites (tri-, mono-, and di-). However, it was apparent that histone H3K4 methylation was lost over generations. In accordance with our findings, H3K4me3 trimethylation has been shown to be transgenerationally transmitted for up to three generations and to be associated with longevity in *Caenorhabditis elegans* (104). Apart from H3K4 methylation, a significant increase in H3K27 methylation, i.e., H3K27me1 and H3K27me3, was also observed. In general, H3K27 methylations are considered as a sign of heterochromatin and is a repressive marker. It is often associated with transcriptional repression or silencing, whereas H3K27me1 are also reported to be associated with active promoters (100, 105, 106). Additionally, some authors have also proposed that H3K4me3 and H3K27me3 can form bivalent domains that can keep regulatory gene expression at very low levels while at the same time keeping them poised for activation (107, 108). This might be a possibility in the current study here, as we observed increased levels of H3K4me3 and H3K27me3 and low levels of immune gene expression. Overall, the present study indicates that exposure of brine shrimp to phloroglucinol induced mostly a state of transcriptionally active chromatin, e.g., H3K4me3, H3K4me2, H3K27me1, total H3 hyperacetylation, and, in some instances, of the gene repression marker, e.g., H3K27me3, H4 hypoacetylation, in the subsequent progenies of brine shrimp. So, histone modifications might have played a role in the phloroglucinol-induced transgenerational-inherited resistant phenotype in brine shrimp.

Phenotypes are made of various genetic and non-genetic components and are often environmentally influenced (109). In transgenerational studies, there is a general interest to examine the presence of associated fitness cost in life history traits such as reproduction. So, in the current study, this possibility was explored. However, neither reproduction cost, nor changes of reproductive phenotypes in the subsequent generations were observed (20, 110), which is beneficial for future applications.

In conclusion, this study reports three major novel findings. At first, phloroglucinol treatment of brine shrimp parents generated transgenerational inheritance of increased resistance against biotic (bacterial challenge) and abiotic stressors (thermotolerance) across three subsequent generations without involving a fitness-related cost. Secondly, some of the innate immune-related genes under study (*DSCAM, proPO, PXN, HSP90, HSP70*, and *LGBP*) displayed a differential expression pattern. Third, there is evidence that epigenetic mechanism such as DNA (5-mC) methylation, RNA (m6A) methylation, and histone modifications (active chromatin marker, i.e., H3K4Me3, H3K4me1, H3K27me1, H3 hyperacetylation, H3K14ac, and repression marker, i.e., H3K27me3, H4 hypoacetylation) might be involved in the acquisition of the resistant phenotype. To the best of our knowledge, this is the first demonstration of transgenerational inheritance of a compound-induced robustness, enhancing protection against both biotic (particularly against AHPND causing bacterial strains *V. parahaemolyticus*) and abiotic stressors (thermotolerance). Therefore, parental conditioning of the brood stock could be a unique and powerful novel strategy for prophylaxis of infectious diseases for future shrimp farming applications. Brine shrimp (*A. franciscana*) is regarded as a model for crustacean shrimp. However, species differ, and further experiments are necessary to find out if our data can be extrapolated to other shrimp species.

## Data Availability Statement

All datasets generated for this study are included in the article/Supplementary Material.

## Author Contributions

SR, PN, PB, and DV conceived the study and designed research. SR and VK performed the experiments. SR analyzed the data and wrote the manuscript. All authors were involved in editing of the manuscript.

### Conflict of Interest

The authors declare that the research was conducted in the absence of any commercial or financial relationships that could be construed as a potential conflict of interest.

## Abbreviations

F0: Parental generation
F1: First filial generation
F2: Second filial generation
F3: Third filial generation
CF0: Control parental generation
CF1: Control first filial generation
CF2: Control second filial generation
CF3: Control third filial generation
TF0: Treatment parental generation
TF1: Treatment first filial generation
TF2: Treatment second filial generation
TF3: Treatment third filial generation
7d: 7th day
16d: 16th day
DAH: Day after hatching
CGT: Common Garden Test
Vp: *V. parahaemolyticus*
Vh: *V. harveyi*.
