# Supplementary material for: Phloroglucinol Treatment Induces Transgenerational Epigenetic Inherited Resistance Against Vibrio Infections and Thermal Stress in a Brine Shrimp (Artemia franciscana) Model
Source: Front Immunol. 2019 Nov 27;10:2745. doi: 10.3389/fimmu.2019.02745 (PMC6890837; doi:10.3389/fimmu.2019.02745)
Supplement: Supplementary file 1 [file Data_Sheet_1.pdf]

Supplementary Material

Table S1: Target immune genes and primer information

		Primers	PCR efficiency	Correlation Coefficient (R^2)	E
**Reference genes**	EF-1a	5’ TCGACAAGAGAACCATTGAAAA 3’<br>5’ ACGCTCAGCTTTAAGTTTGTCC 3’	111.4	0.998	2.129
	GAPDH	5’ GTTGATGGCAAACTCGTCATA 3’<br>5’ CCACCTTCCAAGTGAGCATTA 3’	98.0	0.986	1.960
	**Geomean**				2.043
**Targeted Immune genes**	Hsp70	5’ CGATAAAGGCCGTCTCTCCA 3’<br>5’ CAGCTTCAGGTAACTTGTCCTTG 3’	106.7	0.999	2.067
	Hsp90	5’GCTGACCGTGTTGTTGTCAC 3’<br>5’ ACGATCTTGGTTCCACGTCC 3’	107.5	0.998	2.075
	DSCAM	5’ TCAAGAGGCTGAAAGAGAAGAAAT 3’<br>5’ CAGTAGAAGCAGTGACCCAGAAAT 3’	111.5	0.995	2.114
	LGBP	5’ CCGTGAAGATCCCAACGAAC 3’<br>5’ GGAGGAGGTAATTGGGAGTTTCAAGG 3’	109.5	0.996	2.095
	ProPO	5’ TCTGCAAGGAGGATTTAAGGA 3’<br>5’ TGACTGACAAAGGAGATGGGAC 3’	98.0	0.988	1.976
	Peroxinectin	5’ TTGGTGCTGCTGCTTTTCG 3’<br>5’ CCCCATCGCTTGTCTTCGT 3’	101.5	0.998	2.015
	SOD	5’ CAATCAGCATTGGGGTTTGTC 3’<br>5’ GAATCTCTTCGTTGGTTGTAGGG 3’	100.3	0.996	2.002
	HMGB1	5’AGAGGCGGGAAAGGAAGC 3’<br>5’ CCCACACCAAGACCAGGTTG 3’	109.5	0.992	2.
	Tgase1	5’ GCAAGGAGCTGGAATGGGT 3’<br>5’ TGTTTGGGAGTTAATCGGACTGT 3’	105.4	0.999	2.053

	Tgase2	5’ TTCTTTACACAGGCATTCCGTC 3’<br>5’ GTTACATCAAATCCCAGCTCCA 3’	107.5	0.991	1.973494324

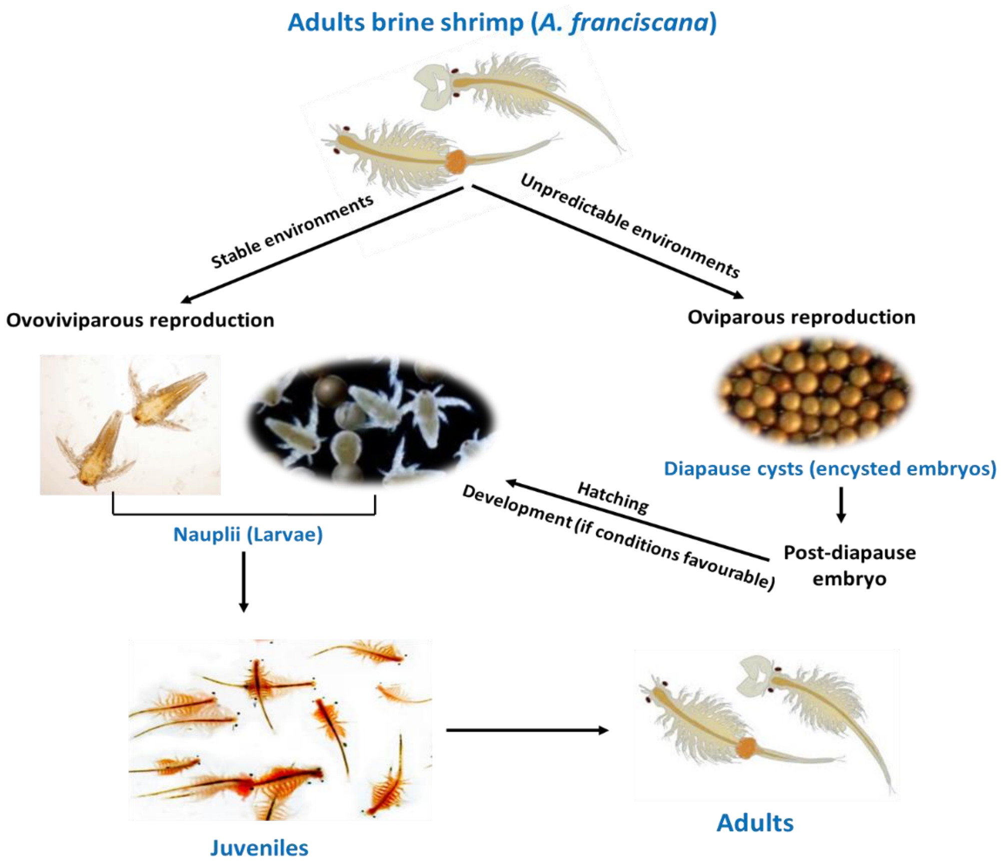

Figure S1: Life cycle of Brine shrimp (*A. fransciscana*)

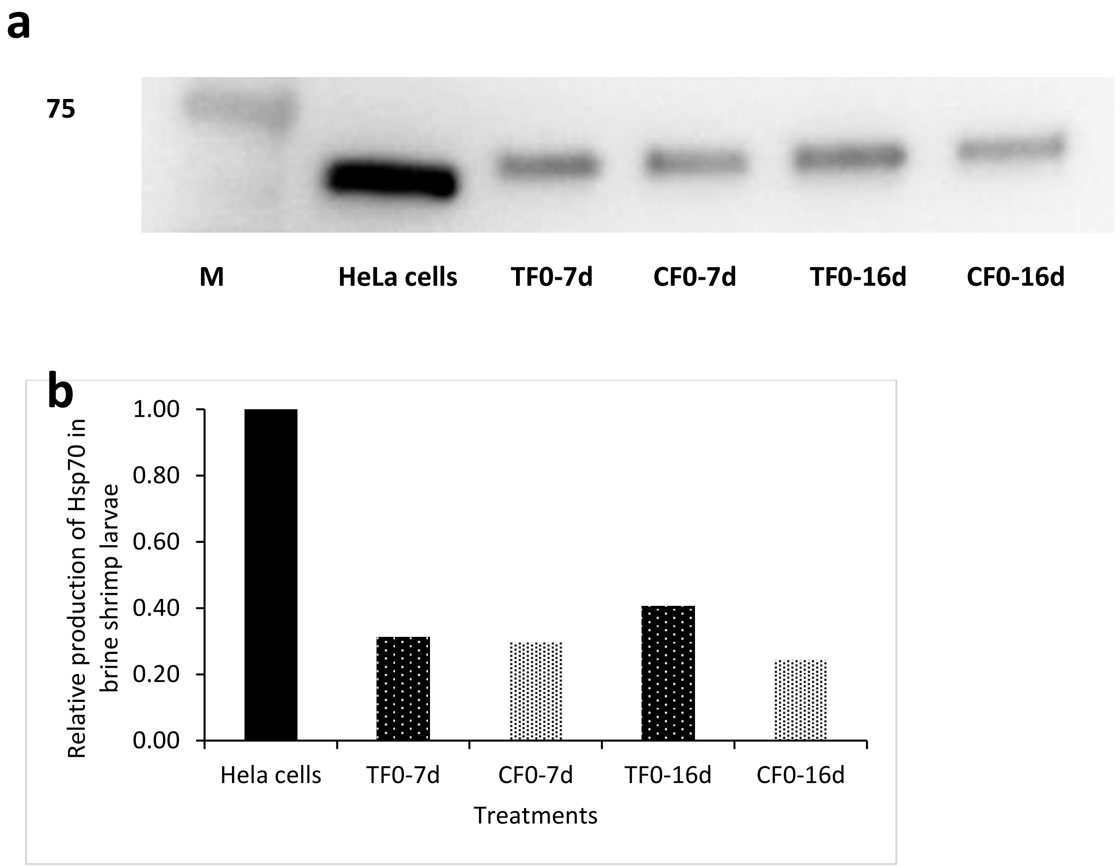

Figure S2: Induction of HSP70 protein in brine shrimp from parental generation 7d and 16d either or not treated with phloroglucinol (2µM). (a) Protein extracted from control and treatment group brine shrimp parents was resolved on an SDS-PAGE gel and then transferred to a polyvinylidene fluoride membrane and probed with an antibody to brine shrimp HSP70. Molecular mass standards (M) in kilodaltons are shown on the left. The induction of HSP70 in HeLa cells was regarded as 1. (b) Quantitative analysis of HSP70 levels in the brine shrimp is presented relative to HSP70 production in HeLa cells.

(a)

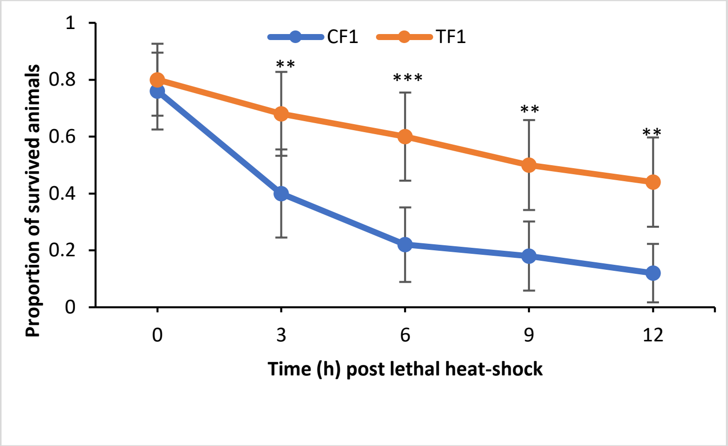
CF1
TF1
Proportion of survived animals
1
0.8
0.6
0.4
0.2
0
**
***
**
**
0
3
6
9
12
Time (h) post lethal heat-shock

(b)

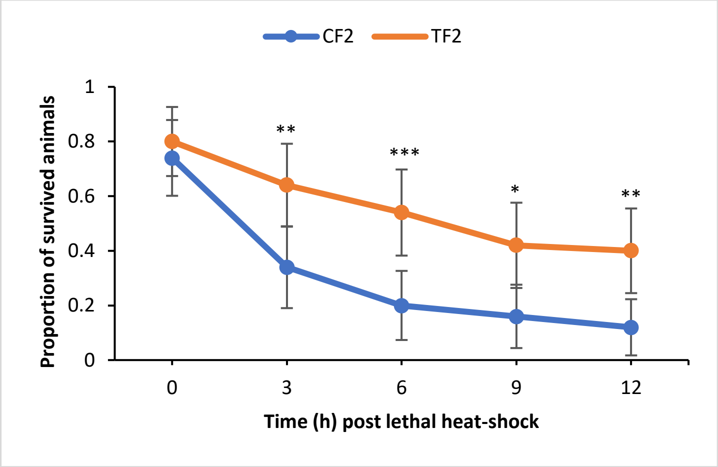
CF2
TF2
Proportion of survived animals
1
0.8
0.6
0.4
0.2
0
**
***
*
**
0
3
6
9
12
Time (h) post lethal heat-shock

(c)

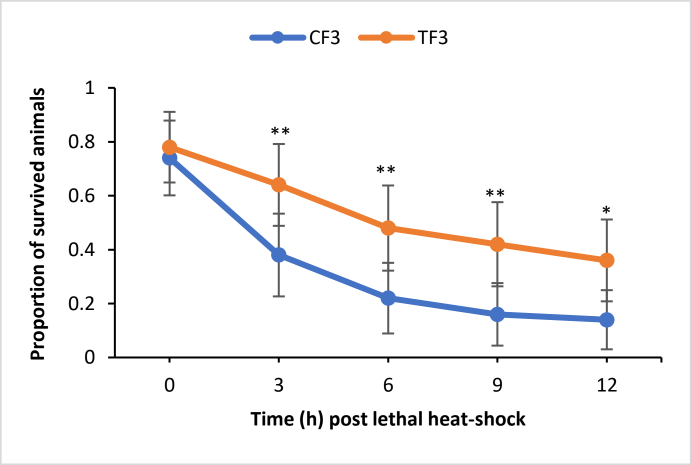
CF3
TF3
Proportion of survived animals
1
0.8
0.6
0.4
0.2
0
**
**
**
*
0
3
6
9
12
Time (h) post lethal heat-shock

Figure S3: Phloroglucinol treatment of parental brine shrimp increased the resistance of their progeny for 3 subsequent unexposed generations against lethal heat shock

Cysts collected from F1, F2 and F3 generations were hatched simultaneously in the common garden test (CGT) and after which the axenic age and size synchronized larvae were used for the lethal heat shock assay. One-day old axenic brine shrimp from both the control and treatment group (F1, F2 and F3 generation) was transferred into separate 40 ml glass tubes containing 20 ml of 35 g/L sterile artificial sea water (20 animals/tube) that were maintained in 5 replicates (biological replicates at the challenge level). Brine shrimp larvae underwent a lethal heat shock treatment at 42.5°C for 15 min, and afterwards were immediately transferred to 28°C. Survival was scored at every 3 h intervals till 12h post lethal heat shock**.** Error bars represent the standard deviation (n=5) and stars represents the significant difference over time at each time point *(P < 0.05), **(P < 0.01), ***(P < 0.001).

(A)

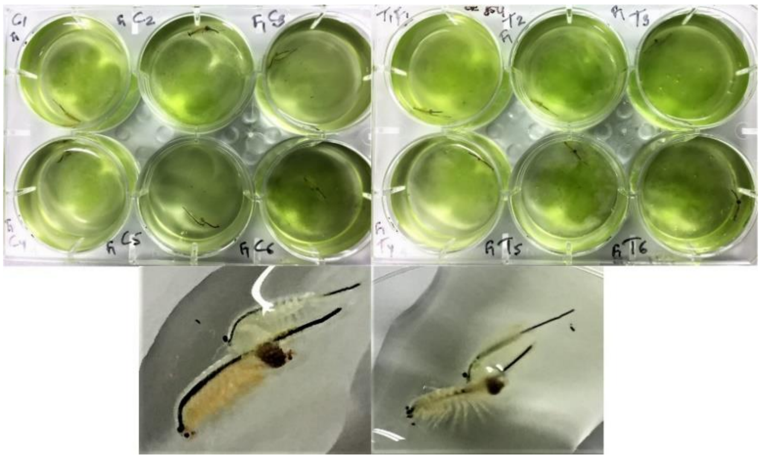

(B)

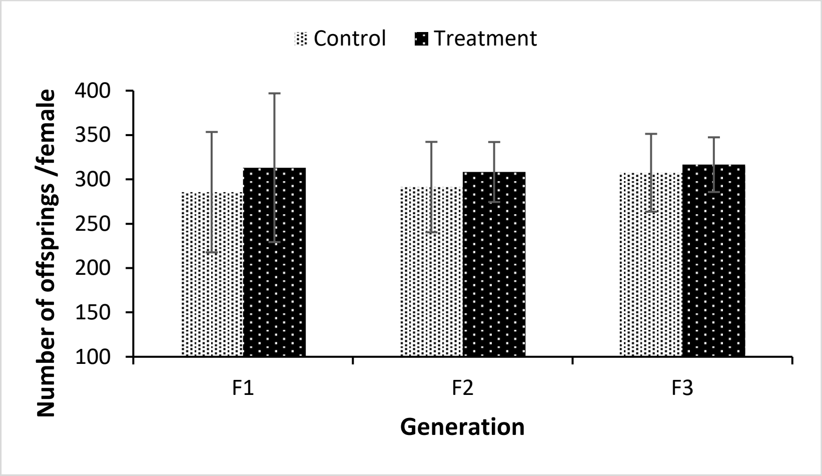

Figure S4: Cost of reproduction and impact of reproductive phenotypes on the brine shrimp. Reproductive performance was assessed in the treatment group compared to respective control progeny in three subsequent F1, F2 and F3 generations. The F1-F3 brine shrimp from both control and treatment group cultured under common garden experiment; when they reached to maturity and started coupling, reproductive phenotypes were assessed based on the fecundity (production of cysts and nauplii). Mating couples (7 pairs) were transferred to individual well in a 6-well plate containing 80 ppt artificial sea water. The pairs were fed daily with green algae, *Tetraselmis suecica*, water containing fecal and excess algal clumps was removed and replaced by an equal amount of fresh 80 ppt artificial sea water and maintained in a controlled room temperature (28°C) with constant illumination of approximately 27 µE/m^{2}s **(A)**. Parental generation exposed to phloroglucinol didn't exhibit significant alteration ($P < 0.05$) of reproductive performance in their successive generations (F1, F2 and F3). The production of total offspring (cyst and nauplii) **(B)** from each couple of

the brine shrimp were monitored twice a week for a period of 3 weeks after their first reproduction. Error bars represent the standard deviation (n=7) and significant difference were checked at $p < 0.05$.

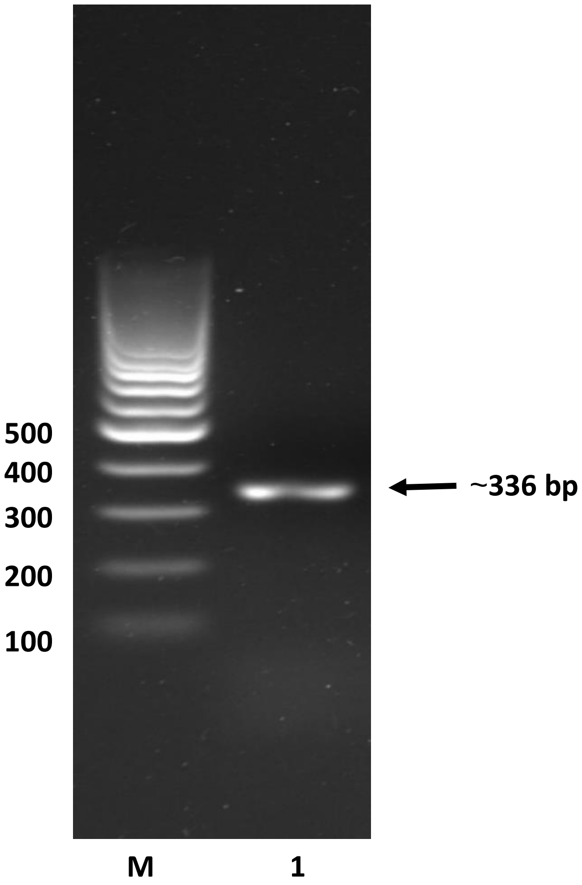

Figure S5: Agarose gel of PCR amplicon from *Vibrio parahaemolyticus* MO904 strain using AP3 method. M - 100 bp DNA ladder, Lane 1- *V. parahaemolyticus* MO904 strain. Positive amplicon (~336 bp) for VPAHPND bacteria from *V. parahaemolyticus* MO904 strain template DNA.

(A) H3K4me3

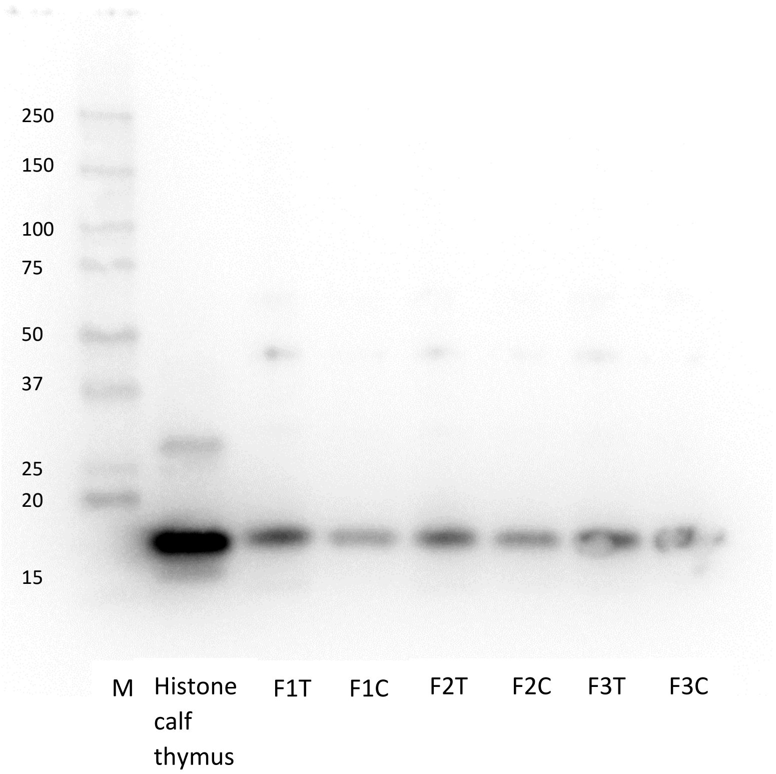

(B) H3K9me3

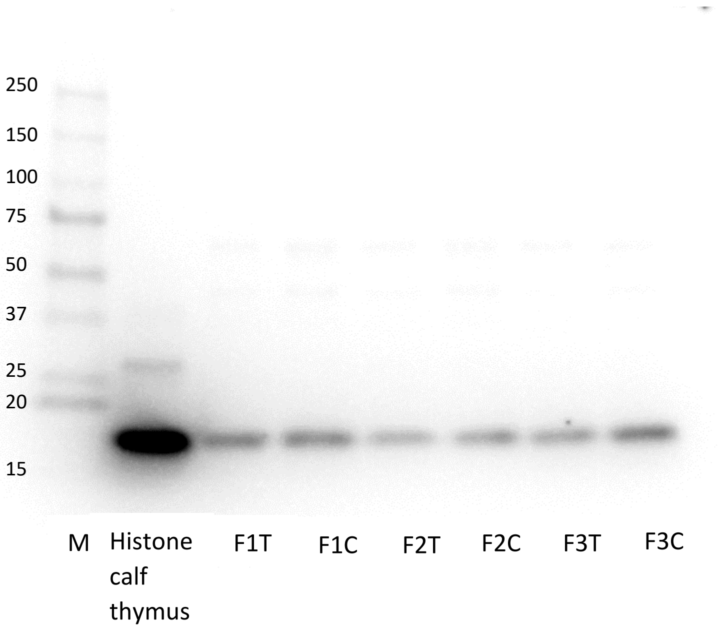

(C) H3K27me3

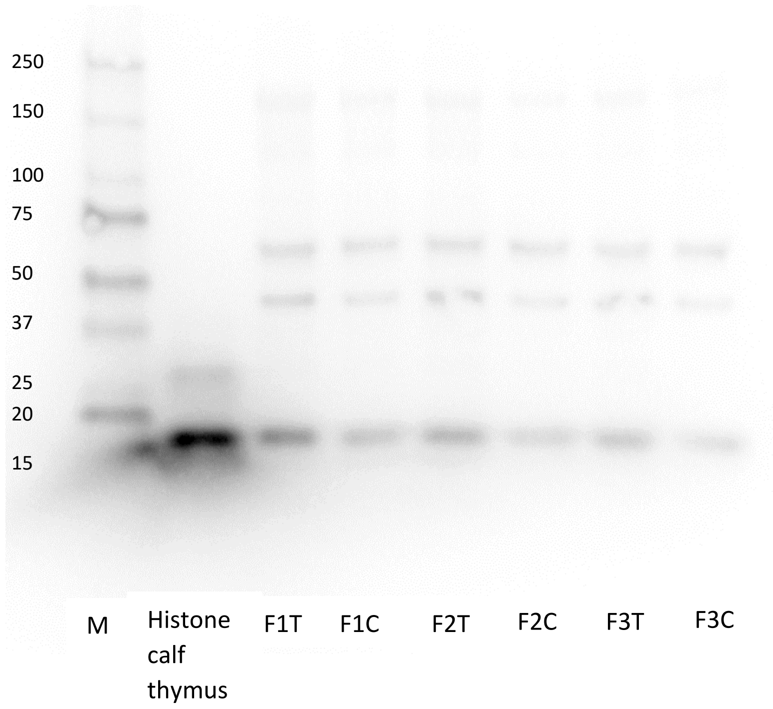

Figure S6: Full immunoblot image were represented for anti-histone H3K4me3, H3K27me3, H3K9me3 and H3K14ac. Histone extracts from the brine shrimp cysts of 3 subsequent generations from treatment and control were resolved on an SDS-PAGE gel and then transferred to a polyvinylidene fluoride membrane and probed with the respective antibody. Molecular mass standards (M) in kilodaltons are shown on the left and standard used here histone calf thymus.

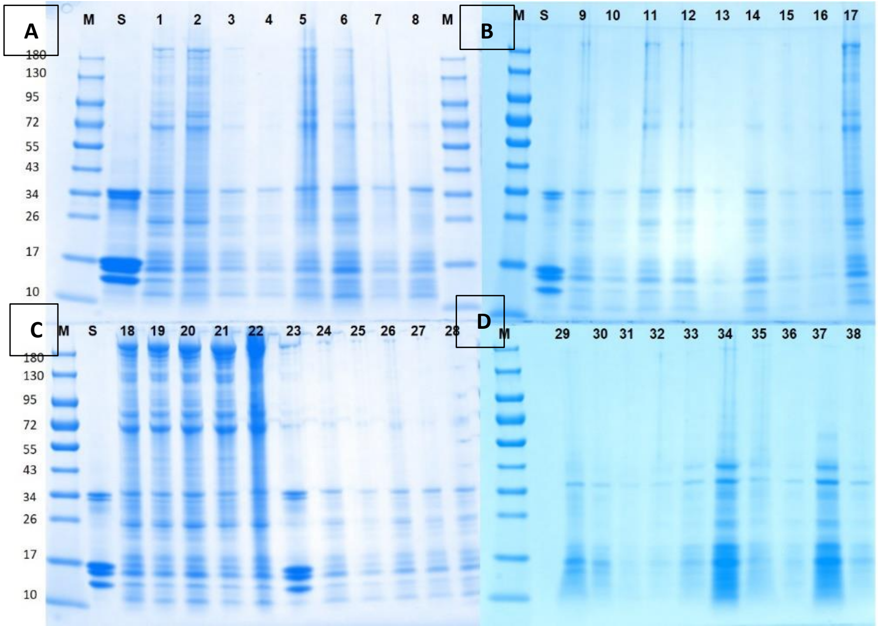

Figure S7: SDS-PAGE analysis for checking the histone quality from brine shrimp (*Artemia franciscana*) cysts and juvenile samples. Histone calf thymus used as standards (S) and Molecular mass standards (M) in kilodaltons (Protein ladder) are shown in figure.
